# Soil Ventilation Benefited Strawberry Growth *via* Microbial Communities and Nutrient Cycling Under High-Density Planting

**DOI:** 10.3389/fmicb.2021.666982

**Published:** 2021-10-18

**Authors:** Yan Zhang, Yujing Hu, Zijing You, Zhenglin Li, Miao Kong, Mingzheng Han, Zhimin Liu, Jie Zhang, Yuncong Yao

**Affiliations:** ^1^Beijing Advanced Innovation Center for Tree Breeding by Molecular Design, Beijing University of Agriculture, Beijing, China; ^2^College of Plant Science and Technology, Beijing University of Agriculture, Beijing, China; ^3^Beijing Key Laboratory for Agricultural Application and New Technique, Beijing, China

**Keywords:** microbial communities, strawberry growth, soil O_2_ and CO_2_, planting density, nutrient cycling, multifunctional index

## Abstract

In order to increase O_2_ concentration in the rhizosphere and reduce the continuous cropping obstacles under high-density cultivation, ventilation is often used to increase soil aeration. Yet, the effect of ventilation on soil microbial communities and nutrient cycling and, further, the extent to which they influence strawberry growth under greenhouse conditions are still poorly understood. Thus, four treatments—no ventilation + low planting density (LD), ventilation + LD, no ventilation + high planting density (HD), and ventilation + HD—of strawberry “Red cheeks” (*Fragaria* × *ananassa* Duch. cv. “Benihopp”) were studied in a greenhouse for 3 years. The ventilation pipe (diameter = 10 cm) was buried in the soil at a depth of 15 cm from the surface and fresh air was sent to the root zone through the pipe by a blower. Ten pipes (one pipeline in a row) were attached to a blower. Soil samples were collected using a stainless-steel corer (five-point intra-row sampling) for the nutrient and microbial analyses. The composition and structure of the soil bacterial and fungal communities were analyzed by high-throughput sequencing of the 16S and 18S rRNA genes, and functional profiles were predicted using PICRUSt and FUNGuild, respectively. The results showed that soil ventilation increased the net photosynthetic rate (Pn), transpiration rate (Tr), and water use efficiency (WUE) of strawberry plants across two growth stages [vegetative growth stage (VGS) and fruit development stage (FDS)]. Soil ventilation increased its available nutrient contents, but the available nutrient contents were reduced under the high planting density compared with low planting density. Both the O_2_ concentration and O_2_:CO_2_ ratio were increased by ventilation; these were positively correlated with the relative abundance of Bacilli, Gamma-proteobacteria, *Blastocatella*, as well as Chytridiomycota and Pezizomycetes. Conversely, ventilation decreased soil CO_2_ concentration and the abundance of Beta-proteobacteria and Gemmatimonadetes. The greater planting density increased the relative abundance of Acidobacteria (oligotrophic group). Ventilation altered soil temperature and pH along with carbon and nitrogen functional profiles in the VGS (more nitrogen components) and FDS (more carbon components), which benefited strawberry plant growth under high planting density. The practice of soil ventilation provides a strategy to alleviate hypoxia stress and continuous cropping obstacles for improving crop production in greenhouse settings.

## Introduction

Over the past century, intensive tillage (such as high-density planting) has often been used in greenhouses to increase the crop yield of per unit land area (Maboko and Du Plooy, 2013). However, long-term use of intensive tillage has major negative impacts on the ecosystem and leads to significant soil degradation, including soil compaction, soil organic carbon (SOC) loss, and essential soil function reduction, which can restrict plant growth and crop yield (Puerta et al., 2018; Stefan et al., 2021).

It is well-known that mesophytic plant roots require soil oxygen (O_2_) in order to respire, grow, develop, and function normally (Li et al., 2016a, b; Ben-Noah and Friedman, 2018). Most of O_2_ supply is obtained directly through diffusion gas exchange from the atmosphere to the rhizosphere soil; meanwhile, the root and microbial respiration is constrained by O_2_ in the root zone (Ityel et al., 2014; Ben-Noah and Friedman, 2018). Under high-density planting condition, reduced O_2_ and elevated carbon dioxide (CO_2_) concentrations negatively affect plant growth and productivity partly (Kitaya et al., 1984; Li et al., 2020). Furthermore, the soil respiration and microbial biomass decreased in most cases of high CO_2_ treatments (Santruckova and Simek, 1997).

Increased O_2_ concentration of root zone can improve bell pepper biomass and fruit yield (Ityel et al., 2014). Meanwhile soil aeration has been reported to accelerate the growth and increase the crop yield by improving O_2_ concentration, such as muskmelon and tomato (Bhattarai et al., 2006; Li et al., 2016, 2020). Thus, aerated irrigation has been widely used in agricultural systems, e.g., orchards, greenhouse, field, and pot experiments (Bhattarai et al., 2004; Friedman and Naftaliev, 2012; Hou et al., 2016; Du et al., 2019). Aerated irrigation promoted the growth of new leaves, fine roots, and new branches of grape, and accelerated air exchange in rhizosphere soil (Zhao et al., 2019). It also has been reported that ventilation (every 3 days) improved leaf functions and increased the net photosynthetic rate (Pn) of young peach trees (Xiao et al., 2015). Thus, soil aeration is considered the third most important factor affecting soil fertility after water and nutrient availability (Glinski and Stepniewski, 1985; Ben-Noah et al., 2021), and its status is an important aspect of soil quality and soil ecology (Niu et al., 2012). Soil aeration elevates the concentration of O_2_ and relieves the high concentrations of CO_2_ formed during respiration under anaerobic soil conditions (Ben-Noah and Friedman, 2018). Tillage is the only commonly used agricultural practice for improving soil aeration, despite its adverse effect on soil structure (Stępniewski and Stępniewska, 2009).

In addition, long-term continuous cropping usually leads to increased soil-borne disease and decreased soil nutrient content of strawberry, which may be caused by the decreased beneficial microorganisms and accumulated fungal pathogen (*Fusarium oxysporum*) in the greenhouse (Huang et al., 2018; Li and Liu, 2019). Intermittent aeration can promote functional bacteria growth in N removal process of wetlands (Liu et al., 2019). Meanwhile, aeration irrigation significantly increased the abundance of aerobic bacteria and promoted the increase of *Pseudomonas* and *Aspergillus* related to phosphate solubilization, that of *Bacillus* related to potassium solubilization, and that of *Fusarium* related to organic matter (OM) decomposition (Zhao et al., 2019).

To alleviate the negative effects of high planting density and continuous cropping, we aimed to improve soil aeration of strawberry through *in situ* ventilation using a blower. Strawberry (*Fragaria ananassa* Duch.) is classified as a plant with high oxygen demand root (Iwasaki, 2008) and is more susceptible to root hypoxic stress. However, few studies are reported that apply root zone aeration as a technique to improve strawberry production (Iwasaki, 2008). Furthermore, the mechanism by which soil aeration may facilitate strawberry growth is largely unknown, especially how it affects soil microbial community composition, and functional structure or alter nutrient concentrations under a high-density planting.

Here, we speculated that *in situ* ventilation is able to influence the soil microbial community, soil nutrient cycling, and their interaction, to benefit the growth of strawberry at high-density planting. To analyze the effect mechanism of soil ventilation on strawberry plant growth, we evaluated the response of soil microorganisms and nutrient content to cultivation management (soil ventilation and planting density treatments) at the strawberry vegetative growth stage (VGS) and fruit development stage (FDS) in greenhouses. The objectives of this study were (i) to evaluate the impact of intermittent soil ventilation on the abundance, diversity, and function of the microbial community under rational high planting density (HD) vs. low planting density (LD) conditions; (ii) to evaluate the impact of intermittent soil ventilation on the soil C and N cycling at the VGS and FDS; and (iii) to evaluate the impact of intermittent soil ventilation on strawberry plant growth.

## Materials and Methods

### Study Site and Plant Materials

The field experiment was conducted in plastic greenhouses in Changping (40°19′ N, 116°16′ E), China, a city located northwest of Beijing. The site lies at an elevation of 57 m above sea level (asl), in a region that has a warm continental monsoonal climate, whose mean temperature ranges from −4 to 26°C, with a mean annual precipitation about 556.4 mm. The soil type here is a typical light sandy loam, with a SOC content of 2.10%, and available N, P, and K concentrations of 143.95, 15.68, and 225.75 mg kg^–1^, respectively. Strawberries were first cultivated at the study site in 2008; hence, by 2016, strawberries have been growing in the area continuously for nearly 10 years. Seedlings of the strawberry “Red cheeks” (*Fragaria* × *ananassa* Duch. *cv.* “Benihopp”) similar in size (i.e., with approximately five compound leaves per plant) were selected and planted in the experimental greenhouses on the ridges in square-shaped rows. The rows were covered with black plastic film. The planting cycle lasts approximately 10 months, from August to May of the next year, amounting to a single growing season with corresponding growth stages for VGS (September to December) and FDS (January to May).

### Experimental Design

Four treatments were set up in the greenhouse: no ventilation + LD, ventilation + LD, no ventilation + HD, and ventilation + HD. The randomized block design was repeated three times, with a total of 12 cultivation plots. The greenhouse was 65.0 m long and 8.0 m wide with an east–west orientation, while each cultivation plot was 5.5 m long and 1.0 m wide with a total planting area of 5.5 m^2^, and crop rows were aligned north–south. The ventilation pipe (diameter = 10 cm, made of nylon fabric and supported with spiral iron wire ring) was buried in the soil at a depth of 15 cm from the surface and fresh air was sent to the root zone through the pipe by a blower (output: 0.75 kw, flow rate: 50 L min^–1^, SCL k05-MS MOR; FBZ, Ferrara, Italy). Ten pipes (one pipeline per row) were attached to a blower, and most air in the pipe can pass through the nylon cloth to the soil (Supplementary Figures 1–3). The ventilation was applied every 2 h and each time lasted for 1 h in 09:00–17:00 during the growing season. The ventilation treatment was administered for 3 years from 2013 to 2016. There are two densities: low planting density (LD, with two plants per row and a plant × row spacing of 10.0 cm × 25.0 cm) vs. high planting density (HD, with four plants per row and a plant × row spacing of 10.0 cm × 12.5 cm) (see Supplementary Figure 1 for details). Every year, seedlings were planted at the end of August and harvested in May of the following year.

### Photosynthetic Characteristics and Leaf Nutrient Content of Strawberry

Leaf photosynthetic activity of strawberry plants was measured using a portable photosynthesis system (LI-6400; LI-COR Biosciences, Lincoln, NE, United States) between 09:00 a.m. and 11:00 a.m. during VGS (November 10, 2015) and FDS (April 10, 2016). Net photosynthetic rate (Pn) and transpiration rate (Tr) were measured and water use efficiency (WUE) was calculated as WUE = Pn/Tr (Ribeiro et al., 2009). Three fully expanded leaves were selected from each plant per plot for the measurements, and average values were calculated for each plant. During the measurements, the photosynthetic photon flux density was 1,200 μmol m^–2^ s^–1^; the CO_2_ concentration in the leaf chamber was 380 ± 5 μmol mol^–1^; and the leaf temperature was 25°C. The leaf area (LA) was measured with a leaf area meter (LI-3000A; LI-COR Biosciences) (Nardini et al., 2014). Leaf N was determined by the Kjeldahl method and leaf P and K were determined by inductively coupled plasma-optical emission spectrometry (ICP-OES) (iCAP 6000; Thermo Fisher Scientific, Waltham, MA, United States) (Rivelli et al., 2012) for 15 leaves per plant that had been oven-dried at 70°C for 24 h.

### Soil Sampling and Processing

Soil samples were collected on two occasions (November 10, 2015 and April 10, 2016). After removing the litter layer, five replicate samples (top 0–20 cm depth) were collected in an “S” shape on the ridge, using a standard soil auger (4.5 cm inner diameter) and then homogenized to obtain a composite sample for each plot. The samples were immediately transported to the laboratory, sieved (<2 mm), and divided into two subsamples. One was immediately stored at −80°C for soil microbial and enzymatic activity analyses; the other portion was air-dried and stored at room temperature until chemically analyzed.

### Soil Physicochemical Parameters

The O_2_ and CO_2_ concentrations in the treated soils were measured with a gas detector (GasAlertMicro 5 IR; BW Technologies Honeywell, Calgary, AB, Canada). Soil temperature was measured with an electronic temperature and humidity recorders (RTR53A; T&D Corporation, Matsumoto, Japan). The pH was measured with a compound electrode (PE 10, Sartorius, Göttingen, Germany) at a soil-to-water ratio of 1:2.5. The SOC content was determined by the Walkley–Black method (Nelson and Sommers, 1996), albeit with some slight modifications. Total nitrogen (TN) content was determined with the Kjeldahl method (Bremner, 1996), and soil available nitrogen (AN) was determined by applying the alkaline hydrolysis diffusion method (Lu, 2000). Total and available phosphorus (TP and AP, respectively), and total and available potassium (TK and AK, respectively) concentrations were measured by ICP-OES (Rivelli et al., 2012). Soil invertase (INV), catalase (CAT), and urease (URE) activity were determined as described by Jin et al. (2009), with some slight modifications applied. To estimate the INV activity, the 3,5-dinitrosalicylic acid method was implemented, using a sucrose substrate, after which 5 g of air-dried soil was incubated at 37°C for 24 h. Catalase activity was measured using the back titration of residual H_2_O_2_ added to soil (2 g) with 0.1 M KMnO_4_. URE activity was determined after incubating the soil (5 g) for 24 h at 37°C with a 10% urea solution as the substrate.

### Soil DNA Extraction and Amplification

From each sample, total genomic DNA was extracted from 0.25 g of soil using the TIANamp Soil DNA Kit (Tiangen Biotech, Beijing, China) according to the manufacturer’s protocol. The quality and quantity of DNA were evaluated based on the A260/280 ratio, measured on a spectrophotometer (NanoDrop 2000; Thermo Fisher Scientific) and by electrophoresis (1% agarose gel). The V3–V4 hypervariable regions of the bacterial 16S rRNA gene was amplified using the barcoded primers 341F (5′-CCTACGGGNGGC WGCAG-3′) and 785R (GACTACHVGGGTATCTAATCC) (Klindworth et al., 2013), and the fungal 18S rRNA gene sequences were PCR-amplified using the barcoded primers EF4 (5′-GGAAGGGRTGTATTTATTAG-3′) and NS2 (5′-GGCTGCTGGCACCAGACTTGC-3′) (Smit et al., 1999; Rashad et al., 2012). The purified Polymerase Chain Reaction (PCR) amplicons were then sequenced on the MiSeq platform (300-bp paired-end reads) (Illumina, San Diego, CA, United States) by Ori-Gene Technology (Beijing, China).

### Soil Microorganisms Sequence Data Analysis

High-quality paired-end reads of the 16S and 18S sequences were merged using the FLASH software (Magoč and Salzberg, 2011) and Mothur^1^ was used to filter the sequences and remove the barcodes. Operational taxonomic units (OTUs) were obtained using the UPARSE pipeline based on the merged sequences (Edgar, 2013); those with ≥97% similarity were assigned to the same OTU. To obtain taxonomic information, representative 16S and 18S sequences of each OTU were generated and aligned to the SILVA and UNITE databases, respectively, using the Ribosomal Database Project (RDP) classifier (Pruesse et al., 2007).^2^ The raw sequences were submitted to NCBI Sequence Read Archive under the identification PRJNA721522.

Alpha-diversity indices, including the number of species observed (Sobs) and the Chao and Shannon indices, were calculated with Mothur v. 1.34.4 (Schloss et al., 2009). The functional groups related to soil C and N cycles of the bacteria were obtained using the Functional Annotation of Prokaryotic Taxa (FAPROTAX) database,^3^ which could map prokaryotic clades to metabolic and ecologically relevant functions based on the literature of cultured strains, by transforming the OUT tables into putative functional profiles (Louca et al., 2016; Xu et al., 2021). Based on the OTUs of fungi, a trophic classification of pathotrophs, saprotrophs, and symbiotrophs was performed using FUNGuild,^4^ as previously described (Nguyen et al., 2016).

### Statistical Analysis

The soil multifunctionality index (SMF) comprises nine soil nutrition indicators and the respective activity of the three enzymes mentioned in the earlier section *Soil Physicochemical Parameters* (Delgado-Baquerizo et al., 2016; Bastida et al., 2017). To obtain the SMF for each plot, individual functions underwent a *Z*-score transformation, and standardized rates of soil functions were then averaged.

The variables were subjected to a three-way analysis of variance (ANOVA). The three factors included in this experimental design were as follows: (i) Ventilation, which had two levels: ventilation and no ventilation; (ii) Density, which had two levels: HD and LD; and (iii) Stage: VGS and FDS. Three-way ANOVA of the abundance of bacterial and fungal populations is provided in the Supporting Information (Supplementary Tables 3, 4, respectively). All data were subjected to one-way ANOVA followed by *post hoc* analyses with Tukey’s Honestly Significant Difference (HSD) test. Differences between the means were considered statistically significant at *p* < 0.05. Different lowercase letters represent significant differences between eight treatments.

Microbial community structure was analyzed by a principal component analysis (PCA) based on relative abundances of the dominant populations at the phylum, class, family, and genus levels. Permutational multivariate (PERM) ANOVA with 9,999 permutations was used to evaluate the influence of factor analysis upon microbial community structure. Redundancy analysis (RDA) was performed to elucidate the relationships between the environmental variables (O_2_, CO_2_, O_2_:CO_2_, temperature, and pH) and the soil chemical factors. Spearman correlation coefficients between soil, plant, and microbial (bacterial and fungal) variables and O_2_ (CO_2_, O_2_:CO_2_, or SMF) were also calculated. Figures were generated using R software v.3.5.3.^5^

## Results

### Photosynthetic Characteristics and Leaf Nutrient Contents of Strawberry

Compared with no ventilation, soil ventilation increased the Pn, Tr, and WUE of strawberry plants in its two growth stages. Pn and WUE were slightly higher in the FDS than VGS, with no significant difference between LD and HD. Soil ventilation increased LA in both VGS and FDS stages. The leaf N concentration was increased in the VGS yet decreased in the FDS, whereas the leaf P and K concentration were both increased by ventilation in both growth stages (Table 1).

**TABLE 1:** Photosynthetic characteristics, leaf area, and nutrient content of strawberry.

Stage	Treatment	Pn (μmol m^–^^2^ s^–^^1^)		Tr (mmol m^–^^2^ s^–^^1^)		WUE (μmol mmol^–^^1^)		LA (cm^2^)		LN (g kg^–^^1^)		LP (mg kg^–^^1^)		LK (mg kg^–^^1^)	
VGS	NVLD	16.72 ± 0.92d		3.77 ± 0.08e		4.44 ± 0.31ab		43.14 ± 3.00c		33.67 ± 1.14cd		287.48 ± 25.99d		962.54 ± 45.53abc	
	VLD	19.94 ± 0.90bcd		4.31 ± 0.09cde		4.63 ± 0.24a		44.21 ± 2.34bc		45.68 ± 1.26a		334.15 ± 15.94cd		970.76 ± 38.46abc	
	NVHD	17.94 ± 0.28cd		4.13 ± 0.27de		4.36 ± 0.34ab		44.56 ± 0.71bc		40.12 ± 1.53b		318.07 ± 21.00cd		873.35 ± 38.03c	
	VHD	20.47 ± 1.03bc		4.49 ± 0.12bcde		4.56 ± 0.23ab		45.10 ± 3.45bc		47.60 ± 1.70a		320.87 ± 15.51cd		925.62 ± 14.27bc	
FDS	NVLD	17.84 ± 1.80cd		5.03 ± 0.23abcd		3.55 ± 0.32b		48.39 ± 3.00bc		31.40 ± 0.60de		419.91 ± 18.21ab		918.43 ± 16.30bc	
	VLD	22.43 ± 0.95ab		5.20 ± 0.63abc		4.35 ± 0.38ab		49.31 ± 2.34bc		29.58 ± 0.72e		454.03 ± 27.61a		1018.94 ± 56.89ab	
	NVHD	19.78 ± 0.26bcd		5.38 ± 0.24ab		3.68 ± 0.13ab		52.01 ± 0.71b		36.25 ± 0.74c		369.16 ± 10.53bc		938.57 ± 15.23bc	
	VHD	25.40 ± 2.21a		5.77 ± 0.44a		4.44 ± 0.69ab		60.94 ± 3.45a		31.59 ± 0.93de		373.61 ± 22.49bc		1066.24 ± 62.37a	

**Significant level**		***F***	***P***	***F***	***P***	***F***	***P***	***F***	***P***	***F***	***P***	***F***	***P***	***F***	***P***

V		64.50	0.000	7.74	0.014	10.75	0.005	23.59	0.000	48.85	0.000	7.00	0.018	19.49	0.000
D		11.21	0.004	7.90	0.012	0.01	0.904	4.24	0.056	66.93	0.000	11.71	0.003	1.05	0.322
S		27.25	0.000	80.77	0.000	11.03	0.004	449.67	0.000	422.44	0.000	114.55	0.000	10.31	0.005
V × D		0.03	0.866	0.01	0.950	0.00	0.966	0.76	0.396	15.70	0.001	4.89	0.042	1.19	0.292
V × S		5.02	0.040	0.43	0.523	3.81	0.069	6.83	0.019	194.69	0.000	0.11	0.748	6.58	0.021
D × S		2.54	0.130	0.52	0.484	0.41	0.533	4.99	0.040	0.67	0.426	19.91	0.000	9.52	0.007
V × D × S		0.75	0.400	0.63	0.447	0.01	0.915	2.33	0.146	0.83	0.377	0.18	0.675	0.07	0.799

*Values are shown as mean ± SD (*n* = 3). Different letters in the same column indicate significant differences between means (*p* < 0.05) (Tukey’s test). D, planting density; FDS, fruit development stage; LA, leaf area; LK, leaf potassium; LN, leaf nitrogen; LP, leaf phosphorus; NVHD, (no ventilation + high planting density); NVLD, (no ventilation + low planting density); Pn, net photosynthesis; S, growth stage; Tr, transpiration rate; V, *in situ* ventilation; VGS, vegetative growth stage; VHD, (ventilation + high planting density); VLD, (ventilation + low planting density); WUE, water use efficiency.*

### Soil Physicochemical Properties

**FIGURE 1:**
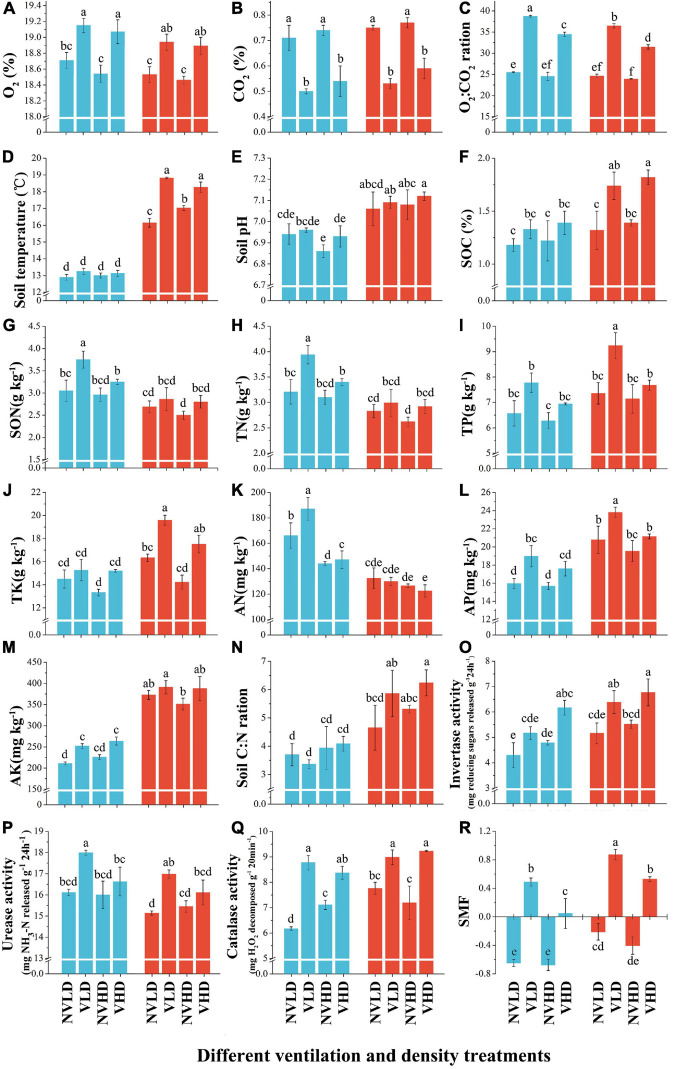
Soil physicochemical index, nutrient index, enzyme activities, and soil multifunctional index (SMF) at two growth stages (vegetative growth stage and fruit development stage) of strawberry under different soil ventilation and planting density treatments. **(A)** O_2_ concentration, %; **(B)** CO_2_ concentration, %; **(C)** O_2_:CO_2_ ratio; **(D)** soil temperature, °C; **(E)** soil pH; **(F)** SOC (soil organic carbon), %; **(G)** SON (soil organic nitrogen), g kg^–1^; **(H)** TN (soil total nitrogen), g kg^–1^; **(I)** TP (soil total phosphorus), g kg^–1^; **(J)** TK (soil total potassium), g kg^–1^; **(K)** AN (soil available nitrogen), g kg^–1^; **(L)** AP (soil available phosphorus), g kg^–1^; **(M)** AK (soil available potassium), g kg^–1^; **(N)** soil C/N ratio (soil organic carbon-to-total nitrogen ratio); **(O)** invertase activity, mg reducing sugars released g^–1^ 24 h^–1^; **(P)** urease (URE) activity, mg NH_3_-N released g^–1^ 24 h^–1^; **(Q)** catalase activity, mg H_2_O_2_ decomposed g^–1^ 20 min^–1^; **(R)** SMF (soil multifunctionality index), mg H_2_O_2_ decomposed g^–1^ 20 min^–1^. The vegetative growth stage (VGS) and fruit development stage (FDS) are shown in sky blue and red, respectively. Results are shown as mean ± SD. Error bars represent standard deviation (SD) (*n* = 3). Different letters indicate significant differences between the means of each index (*p* < 0.05, Tukey’s test).

Soil ventilation significantly increased the soil O_2_ concentration and O_2_:CO_2_ ratio, while reducing the soil CO_2_ concentration. It also increased soil temperature in the FDS, but had no significant effect on soil pH in either growth stage. Planting density significantly influenced the soil O_2_:CO_2_ ratio, in that it was reduced when a greater planting density of strawberry was used. Significant differences in soil properties (soil O_2_, CO_2_ concentrations, O_2_:CO_2_ ratio, temperature, and pH) were evident between the two growth stages (Figures 1A–E and Supplementary Table 1).

Soil ventilation and plant density influenced the levels of most soil nutrients in the two growth stages of strawberry, as seen in Figure 1 and Supplementary Table 2. Soil ventilation increased the SOC content, and this effect was significant in the FDS. It also increased soil organic nitrogen (SON), TN, TP, TK, AP, and AK contents, all of which were diminished by a higher planting density. Soil TN content was higher in the VGS than FDS, while soil TP and TK contents showed the opposite trend (Figures 1G–J).

Soil ventilation enhanced the activities of three soil enzymes (URE, INV, and CAT). The INV activity was increased with a greater planting density. URE activity was slightly higher while INV activity was lower in the VGS than FDS (Figures 1O–Q). Soil ventilation increased the SMF, and it was higher in the FDS than VGS (Figure 1R and Supplementary Table 2).

**FIGURE 2:**
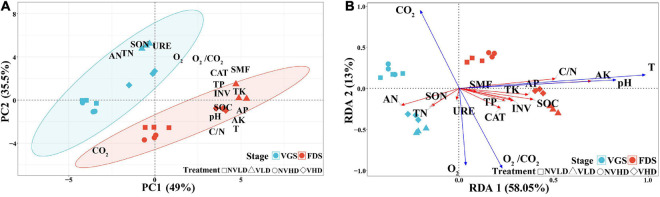
Principal component analysis (PCA) based on soil chemical and physicochemical parameters as variables and redundancy analysis (RDA) of the soil chemical parameters data as explained by environmental variables (O_2_, CO_2_, O_2_/CO_2_, T, and pH) at two growth stages (vegetative growth stage and fruit development stage) of strawberry under different soil ventilation and planting density treatments. **(A)** PCA; **(B)** RDA. The vegetative growth stage (VGS) and fruit development stage (FDS) are shown in sky blue and red, respectively.

The PCA revealed significant differences between the two growth stages of strawberry plants. The soil N cycle dominated in the VGS whereas the soil C cycle (including the P and K cycles) was dominated in the FDS (Figure 2A). The RDA showed that soil O_2_ concentration was positively correlated with those soil nutrient variables related to N cycling; soil temperature and pH were redundant among the soil nutrient variables that are related to C cycling (Figure 2B).

### Diversity, Composition, and Structure of Soil Bacterial Community

**FIGURE 3:**
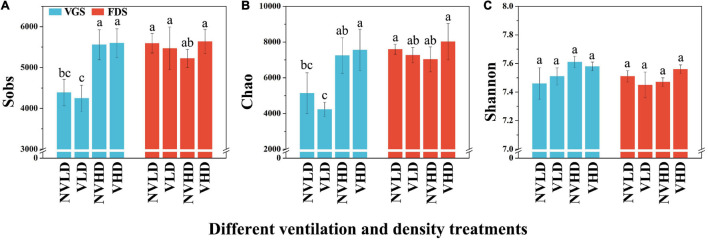
Sobs **(A)**, Chao **(B)**, and Shannon **(C)** indices of the bacterial community in the studied soils. The vegetative growth stage (VGS) and fruit development stage (FDS) are shown in sky blue and red, respectively. Sobs is the observed operational taxonomic units (OTUs); Chao was used to evaluate community richness based on OTUs; the Shannon index was used to assess community diversity, considering the contributions of rare taxa. Results are shown as mean ± SD. Error bars represent standard deviation (SD) (*n* = 3). Different letters indicate significant differences between the means of each index (*p* < 0.05, Tukey’s test).

The Sobs, Chao, and Shannon indices for the total bacterial community, estimated in terms of its OTUs and relative abundances, were not significantly affected by ventilation according to the PERMANOVA results (Supplementary Table 3). However, the bacterial diversity (Shannon index) and richness were higher at HD than LD in the VGS, but there was no significant difference at the FDS (Figure 3).

**FIGURE 4:**
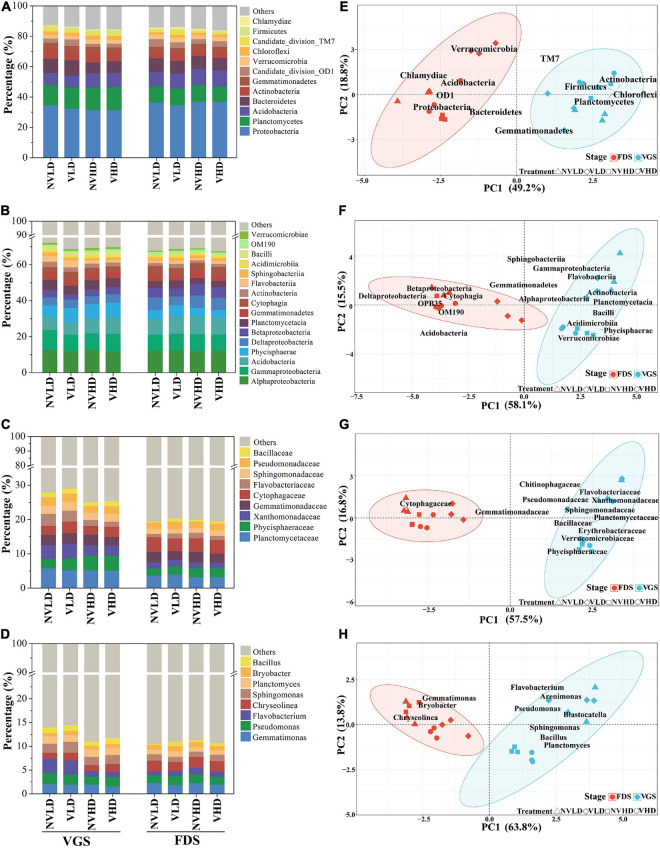
Total bacterial community composition as determined by 16S rRNA gene sequencing. **(A–D)** Community composition at the phylum **(A)**, class **(B)**, family **(C)**, and genus **(D)** levels. **(E–H)** Community structure at the phylum **(E)**, class **(F)**, family **(G)**, and genus **(H)** levels. Only dominant populations (as long as one of the treatments has a relative abundance > 1%) are represented. The vegetative growth stage (VGS) and fruit development stage (FDS) are shown in sky blue and red, respectively.

The soil bacterial community was dominated by the following phyla: Proteobacteria (34%), Planctomycetes (12%), Acidobacteria (9%), Bacteroidetes (8%), and Actinobacteria (5%). Ventilation markedly reduced the relative abundance of Proteobacteria, Gemmatimonadetes, and Chloroflexi, and increased that of Firmicutes. Compared with LD, a greater planting density (HD) increased the relative abundance of Acidobacteria but decreased that of Bacteroidetes in the VGS. The significant differences were found between the two growth stages of strawberry. For example, the relative abundances of Proteobacteria, Acidobacteria, and Chlamydiae were all lower while those of Planctomycetes, Actinobacteria, Chloroflexi, and Firmicutes were higher in the VGS than FDS (Figure 4A). The dominant phyla were significantly influenced by the interaction term of ventilation × density.

The relative abundances of the top 16 dominant classes of bacteria were influenced by ventilation and planting density at the two growth stages. Ventilation altered the relative abundances of seven classes, with increases in Phycisphaerae and Bacilli and decreases in Alphaproteobacteria, Betaproteobacteria, Deltaproteobacteria, and Gemmatimonadetes. Planting density markedly influenced the relative abundances of 11 classes: in this respect, Acidobacteria and Verrucomicrobiae were higher whereas Flavobacteriia and Sphingobacteriia were lower at the HD than LD in the VGS (Figure 4B). Ventilation significantly increased the relative abundance of Phycisphaeraceae in the VGS and Bacillaceae in the FDS, though it reduced that of Gemmatimonadaceae in the FDS (Figure 4C and Supplementary Table 4C). The relative abundance of *Bacillus* was increased by soil ventilation whereas that of *Pseudomonas* and *Flavobacterium* was reduced by higher planting density at the VGS (Figure 4D).

The PERMANOVA revealed significant effects of ventilation, density, and growth stage, as well as their interactions, on microbial community structure at different taxonomic levels (Supplementary Table 5). Ventilation, mainly affected the structure of the total bacterial community at family and genus levels, while planting density did so at the class and genus levels. Significant differences between the two growth stages arose mainly at the genus level (Figures 4E–H and Supplementary Table 5).

### Diversity, Composition, and Structure of Soil Fungal Community

**FIGURE 5:**
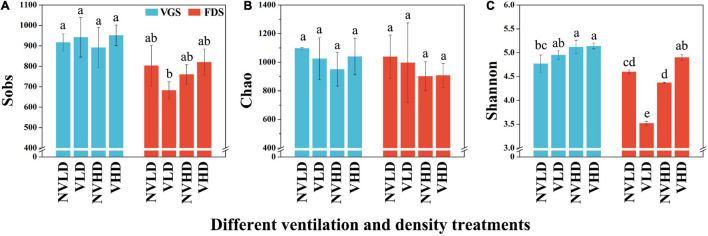
Sobs **(A)**, Chao **(B)**, and Shannon **(C)** indices of the fungal community in the studied soils. The vegetative growth stage (VGS) and fruit development stage (FDS) are shown in sky blue and red, respectively. Sobs is the observed operational taxonomic units (OTUs); Chao was used to evaluate community richness based on OTUs; the Shannon index was used to assess community diversity, considering the contributions of rare taxa. Results are shown as mean ± SD. Error bars represent standard deviation (SD) (*n* = 3). Different letters indicate significant differences between the means of each index (*p* < 0.05, Tukey’s test).

The Sobs, Chao, and Shannon indices of the total fungal community were also not significantly affected by ventilation (Supplementary Table 3). The fungal diversity (Shannon index) was influenced by planting density and growth stage of strawberry plants and interactions among the three factors, being lower in the FDS than VGS (Figure 5 and Supplementary Table 3).

**FIGURE 6:**
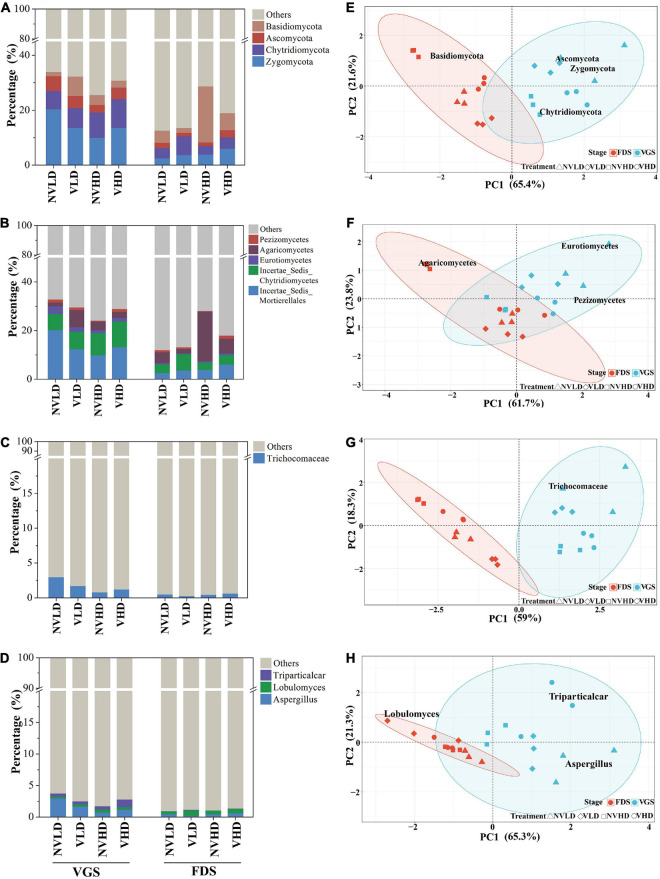
Total fungal community composition as determined by 18S rRNA gene sequencing. **(A–D)** Community composition at the phylum **(A)**, class **(B)**, family **(C)**, and genus **(D)** levels. **(E–H)** Community structure at the phylum **(E)**, class **(F)**, family **(G)**, and genus **(H)** levels. Only dominant populations (as long as one of the treatments has a relative abundance > 1%) are represented. The vegetative growth stage (VGS) and fruit development stage (FDS) are shown in sky blue and red, respectively.

The fungal community was dominated by Zygomycota (9%), Chytridiomycota (6%), Basidiomycota (6%), and Ascomycota (3%) in all four treatment groups (Figure 6A). Ventilation reduced the relative abundance of Basidiomycota and increased that of Chytridiomycota. The relative abundance of Basidiomycota was higher, whereas that of Ascomycota and Zygomycota were both lower at the HD than LD. Furthermore, the interactions of the three factors influenced the relative abundance of the fungal community to different degrees at the phylum level (Figure 6A and Supplementary Table 6).

The relative abundances of three dominant classes of fungi were altered by the treatments. For instance, soil ventilation reduced the relative abundance of Agaricomycetes and increased that of Pezizomycetes. The relative abundance of Eurotiomycetes and Pezizomycetes was higher, while that of Agaricomycetes was lower in the VGS than in the FDS (Figure 6B). *Aspergillus*, *Lobulomyces*, and *Triparticalcar* were the three dominant genera in soil. The relative abundance of *Aspergillus* was affected by density and growth stage and their interactions; that of *Lobulomyces* was increased by ventilation in the FDS (Figure 6D).

### Functional Diversity of Microbial Community

#### Functional Groups Related to the Soil C Cycle

**FIGURE 7:**
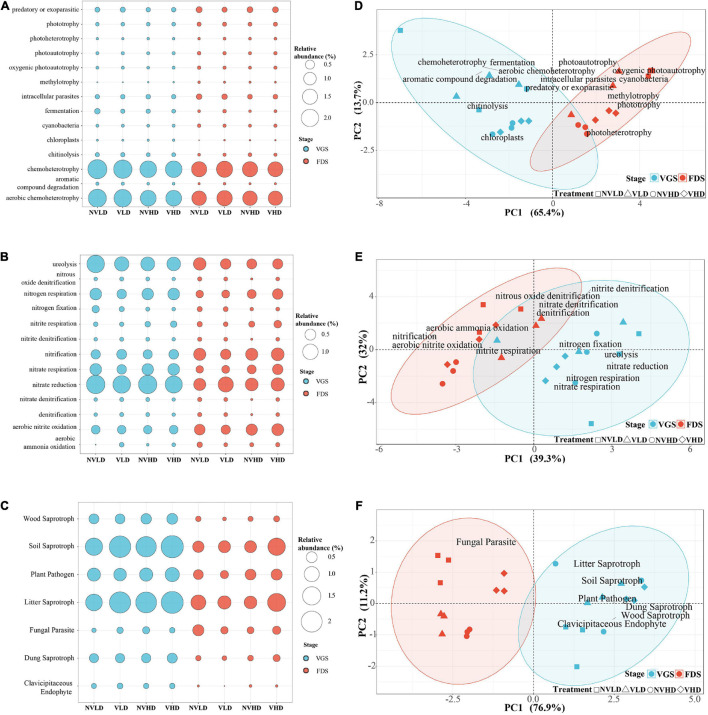
Composition and structure of the functional groups in microbial communities. **(A–C)** Composition of the bacterial community related to the C cycle **(A)** and N cycle **(B)** and of the fungal community **(C)**. **(D–F)** Functional structure of the bacterial community related to the C cycle **(D)** and N cycle **(E)** and of the fungal community **(F)**. The vegetative growth stage (VGS) and fruit development stage (FDS) are shown in sky blue and red, respectively.

Among the 14 functional groups related to the C cycle in the bacterial community identified by FAPROTAX analysis, chemoheterotrophy and aerobic chemoheterotrophy assemblages were dominant. Ventilation reduced the enrichment of predatory or exoparasitic and methylotrophy groups. However, enrichment of the chemoheterotrophy, aerobic chemoheterotrophy, intracellular parasites, photoautotrophy, cyanobacteria, oxygenic photoautotrophy, and aromatic compound degradation functional groups all decreased with an increased planting density. Growth stage influenced the assemblage of 13 observed functional groups, including chemoheterotrophy, chitinolysis, and aromatic compound degradation, all of which were higher in the VGS than FDS, and *vice versa* for the photoautotrophy, cyanobacteria, and methylotrophy trends (Figure 7A).

#### Functional Groups Related to the Soil N Cycle

Functional groups related to the N cycle in the bacterial community indicated that the dominant assemblages of bacterial groups were associated with nitrate reduction, ureolysis, aerobic nitrite oxidation, nitrate respiration, N respiration, nitrification, N fixation, and nitrite respiration in the studied soils. Ventilation inhibited the assemblage of the ureolysis group yet promoted that of the nitrite respiration group. When compared with LD, HD reduced the assemblage of ureolysis and N fixation groups, but it increased that of the nitrification, nitrate respiration, and nitrite respiration groups. Growth stage influenced the assemblage of eight functional groups related to N cycle (Figure 7B and Supplementary Table 3).

#### Functional Diversity of Fungal Community

Functional groups identified by FUNGuild analysis in the fungal community showed that the dominant assemblages in soil were litter saprotroph, soil saprotroph, and plant pathogen. Ventilation significantly reduced the assemblages of fungal parasite groups and enhanced that of soil saprotrophs, which were increased by the HD (Figure 7C).

The PCA of the relative abundances of bacterial and fungal functional groups revealed that they sorted according to the growth stage (S) of strawberry along PC1 (Figures 7D–F and Supplementary Table 8).

### Correlations Between Soil Ventilation and Measured Variables

**FIGURE 8:**
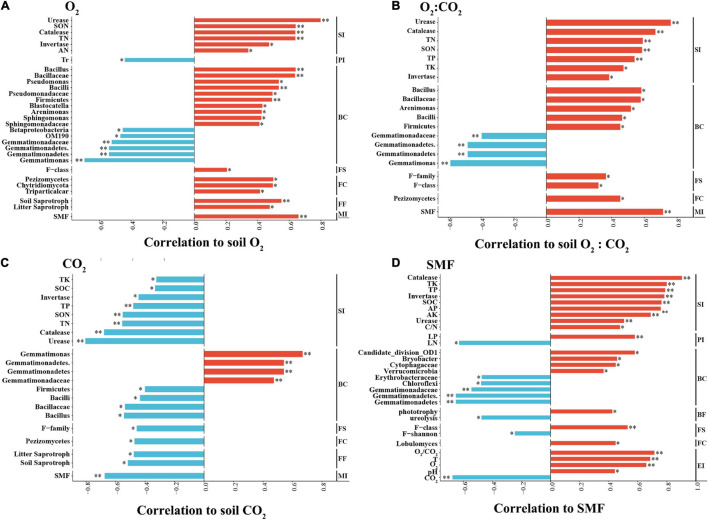
Spearman correlation coefficients between soil environmental factors and soil physicochemical parameters and microbial community. **(A)** Soil O_2_ concentration; **(B)** soil CO_2_ concentration; **(C)** soil O_2_:CO_2_ ratio; **(D)** SMF (soil multifunctionality index). The abundance of total bacterial and fungal populations was analyzed by their 16S and 18S rRNA gene sequencing, respectively. BC, bacterial composition; BF, bacterial function; BS, bacterial structure; FC, fungal composition; FF, fungal function; FS, fungal structure; MI, multifunctional index; PI, plant index; SI, soil index; EI, environmental index. The negative and positive correlations are shown in sky blue and red, respectively. ^∗^*p* < 0.05; ^∗∗^*p* < 0.01.

Soil O_2_ concentration was positively correlated with soil N components. Importantly, it was positively correlated with the relative abundances of Firmicutes (Bacilli, Bacillaceae, and *Bacillus*); Pseudomonadaceae (*Pseudomonas*); *Arenimonas*, Blastocatella, Sphingomonadaceae, *Sphingomonas*, Chytridiomycota (Triparticalcar), and Pezizomycetes, but negatively correlated with those Betaproteobacteria and Gemmatimonadetes (Gemmatimonadetes, Gemmatimonadaceae, and Gemmatimonas). Soil O_2_ concentration was positively correlated with litter and soil saprotrophs in the fungal community, but not correlated with the C and N functional groups of the bacterial community. Additionally, soil O_2_ concentration was negatively correlated with the Tr of strawberry (Figure 8A).

For soil CO_2_ concentration, the pattern of its correlations with the above indices was the opposite of that found for soil O_2_ concentration. More specifically, soil CO_2_ concentration was negatively correlated with the SOC content and TP and TK concentrations (Figure 8B). Soil temperature and pH were negatively correlated with soil CO_2_ concentration. SMF was positively correlated with O_2_ concentration and with the relative abundances of Verrucomicrobia and *Bryobacter*, yet negatively correlated with those of Chloroflexi and Gemmatimonadetes, as well as with the functional group capable of ureolysis. Additionally, the SMF was positively correlated with the leaf P concentration of strawberry plants (Figure 8D).

## Discussion

### Soil Ventilation Effects on the Profile of Soil Microbial Community

In this study, *in situ* ventilation increased the concentration of soil O_2_ and decreased that of soil CO_2_ (Figure 1). O_2_ concentration and supply rates in the root zone plays a critical role in root respiration, root elongation, nutrient absorption and transpiration, and plant growth (Zhao et al., 2019). In this process, soil bacterial and fungal communities also play vital roles in the turnover of SOC and C cycling (Zhao et al., 2018). In the present work, OM decomposition driven by the soil microbial community was activated by ventilation. It has been reported that soil microbial communities regulate many ecosystem processes and contribute to nutrient cycling through the decomposition of OM (Bender et al., 2016; Hartman et al., 2018). Moreover, the positive (soil O_2_) and negative (soil CO_2_) correlation with soil N components suggest that soil O_2_ and CO_2_ have antagonistic effects on N mineralization through soil enzyme activities. This may be consistent with the fact that rhizosphere ventilation can improve the potted tomato root zone environment, increase the soil enzyme activity, and promote the nutrient uptake (Niu et al., 2012).

In this study, ventilation increases relative abundance of Firmicutes phyla and its Bacilli (class, family, and genus) and decreases that of Gemmatimonadetes (Figure 4). It has been reported that aeration irrigation significantly increased the abundance of aerobic bacteria, such as *Nitrospira* and *Cytophagia* (Zhao et al., 2019). Furthermore, the abundance of *Bacillus* was positively correlated with soil O_2_ concentrations (Figure 8A). Bacteria of the genera *Pseudomonas* and *Bacillus* can promote plant growth and protect plants from pathogens. A high relative abundance of aerobic *Bacillus* promotes plant growth and inhibits the growth of pathogenic bacteria *via* the decomposition of labile OM in soil (Razanamalala et al., 2018; Molina-Santiago et al., 2019). Conversely, Gemmatimonadetes abundance was negatively correlated with soil O_2_ concentration (Figure 8A). This result is consistent with the finding that the relative abundance of Gemmatimonadetes decreased in the presence of wheat residues (Bernard et al., 2007) and that Gemmatimonadetes was more active in soils without leaf litter than in the litter treatments (Pfeiffer et al., 2013). Thus, members of the Gemmatimonadetes phylum may be adapted to a lifestyle associated with OM sources that are challenging to mineralize along O_2_ gradients (Whitman et al., 2016).

Ventilation did not alter the fungal alpha-diversity in soil (Supplementary Table 3), but enhanced Chytridiomycota and reduced Basidiomycota at the phylum level, reduced Pezizomycetes and Agaricomycetes at the class level, and increased *Triparticalcar* at the genus level (Figure 6). The OM decomposition rate was positively correlated with the relative abundance of Chytridiomycota, perhaps because of the association between the availability of labile C and the soil O_2_ concentration, which is related to the recruitment of Chytridiomycota (Procter et al., 2014). The relative abundance of Basidiomycota decreased with soil O_2_ concentration, suggesting that wood decay and plant litter decomposition were slowed in the ventilated soil. Ventilation increased abundances of litter and soil saprotrophs groups, which were positively correlated with soil O_2_ concentrations (Figure 8), which may promote the decomposition of soil labile OM and inhibit the occurrence of plant pathogens, resulting in accelerated N mineralization rates (Razanamalala et al., 2018). It also has been demonstrated that although some fungi are sensitive, the community remains stable upon changes in soil O_2_ or CO_2_ (Kaisermann et al., 2014; Zhang et al., 2016; Zi et al., 2018). Thus, aerobic bacteria and functional fungi of soil play major roles in accelerating N mineralization under ventilated conditions.

### Planting Density Effects on Soil Oligotrophic and Copiotrophic Groups

The crop cultivation model can influence soil microbial attributes (Hartmann et al., 2015; García-Delgado et al., 2019; Xiao et al., 2019). For example, intra- and inter-specific plant competition, especially at a high planting density, as well as the identity of the plant species themselves, can influence the rhizosphere bacterial community (Cavalieri et al., 2020). In our study, the planting density affected soil CO_2_ concentration, and the TN and AN concentration and URE activity were all higher at LD when compared to HD in the VGS (Figure 1). Planting density has been shown to alter soil nutrient supply in the forest: increased N mineralization was beneficial to plant morphogenesis and increased the N content of leaves, while C mineralization enhanced plant C assimilation and water utilization and increased the leaf P content (Bastida et al., 2017). Consistent with those results for soil nutrients, compared with HD, we also found that the relative abundances of oligotrophic groups (Acidobacteria and Chloroflexi) were lower while those of copiotrophic groups (Bacteroidetes, Actinobacteria, and Gammaproteobacteria) were higher at LD. Similar results were reported recently, in that Proteobacteria was more abundant under a high-intensity thinning treatment, whereas Acidobacteria was more abundant under low-intensity thinning and control treatments (Dang et al., 2018). The changes to copiotrophic and oligotrophic groups according to planting density suggests that the soil microbial community composition depends on soil nutrient levels, further indicating a cooperative effect among microbial communities in adapting to the external environment. This is because N inputs significantly increased the relative abundance of the predicted copiotrophic groups (Proteobacteria and Firmicutes) but reduced those of predicted oligotrophic groups (Acidobacteria, Nitrospirae, and Chloroflexi) (Ling et al., 2017). By contrast, HD reduced the enrichment of soil functional groups related to C (cyanobacteria, photoautotrophy) and N (ureolysis, N fixation) cycling. These functional groups may be sensitive to high C input in aerated soil, resulting in decreased C and N contents in aerated HD soil, because of soil resource competition during plant growth (Dang et al., 2018; Muñoz-Rojas et al., 2018). Soil, litter, and dung saprotrophs were enriched at HD, and this probably accelerated the C and N cycling dynamics. It is likely that LD slowed the degradation of organic substances due to low C input and reduced competition for nutrients between plants and microbes (Dang et al., 2018; Mommer et al., 2018).

### Growth Stage Effects Soil Nutrient Composition *via* Soil Temperature and pH

Soil temperature and pH were both higher in FDS than in VGS during the strawberry growth stage (Figure 1). They are important environmental factors influencing soil microflora (Cheng et al., 2017; Lammel et al., 2018; Walker et al., 2018). These caused significant differences in the soil C and N compositions and enzyme activities in the ventilation soil. Usually, soil temperature and pH also co-vary with some confounding factors, such as land use and management practices (e.g., tillage, fertilizer application), plant cover, and climatic conditions. We found that soil temperature and pH play important roles in shaping the soil nutrient composition to meet the stage requirements for plant growth and development in ventilated soil. A more comprehensive analysis of the direct (e.g., Actinobacteria) and indirect effects of pH can clarify the mechanisms that shape soil microbial communities (Lammel et al., 2018). In this study, the soil N cycle was dominant in the VGS whereas the soil C cycle (accompanied by P and K cycles) was dominant in the FDS (Figure 2A), with corresponding trends found for the functional groups related to soil C and N cycles. Temperature-induced differences in gross N flux are related to total C availability and the soil microbiome profile (Cookson et al., 2007). Rhizospheric microbial community profiles of a variety of plants (e.g., pea, wheat, sugar beet, and alfalfa) are altered according to the plant developmental gradient (Mougel et al., 2006; Houlden et al., 2008); for example, the root microbiota of rice varies over time during the life cycle of rice plants in the field (Zhang et al., 2018). This potential selection of microbes in the rhizosphere through plant aging may be associated with the ability of beneficial microbes to adapt to intrinsic requirements through plant growth and development by promoting systemic tolerance to abiotic stress, increasing plant’s innate immunity (Zamioudis and Pieterse, 2012), and enhancing mineral nutrition (van der Heijden et al., 2008).

## Conclusion

Our study shows that soil ventilation altered the microbial community profile and drove differences in C and N contents between VGS (more N components) and FDS (more C components), which enhanced strawberry growth under high-density planting environment. The soil aerobic bacteria (e.g., *Bacillus*) and functional fungi (e.g., litter and soil saprotrophs groups) play a major role in accelerating N mineralization under ventilation treatments. The copiotrophic and oligotrophic groups seem to cooperate to benefit strawberry growth mediated by planting density. However, further and more investigation of copiotrophic and oligotrophic bacterial groups are asked to test this hypothesis. More N components should be supplied in the VGS while both C and N components should be applied in the FDS to meet the requirements for normal strawberry plant and fruit development. Soil ventilation can be used as a tillage practice for improving soil quality, keeping soil health and maximize crop production in greenhouse ecosystems with high-density planting (hypoxia stress) or long-term continuous cropping. In order to reveal the underlying mechanism, the effect of ventilation on soil aggregate and functional genes of soil microbial community participating in N mineralization require further study. Meanwhile, it is crucial to consider the soil aeration and their effect on the whole soil microbiome when planning optimal agricultural management practices in greenhouse.

## Data Availability Statement

The microbiota sequencing data presented in the study are deposited in the National Center for Biotechnology Information (NCBI) repository, accession number PRJNA721522.

## Author Contributions

YY and JZ designed the study. YZ, YH, ZY, and ZLL performed the greenhouse and laboratory experiments. ZLL, MH, and ZML performed the data analysis. YZ, JZ, and YY wrote the first draft of the manuscript. All the authors contributed to the writing of this manuscript and approved the final version.

## Conflict of Interest

The authors declare that the research was conducted in the absence of any commercial or financial relationships that could be construed as a potential conflict of interest.

## Publisher’s Note

All claims expressed in this article are solely those of the authors and do not necessarily represent those of their affiliated organizations, or those of the publisher, the editors and the reviewers. Any product that may be evaluated in this article, or claim that may be made by its manufacturer, is not guaranteed or endorsed by the publisher.

## Footnotes

1
https://mothur.org

2
https://rdp.cme.msu.edu/

3
www.zoology.ubc.ca/louca/FAPROTAX

4
https://github.com/UMNFuN/FUNGuild

5
https://www.r-project.org/
