# Supplementary figures and images for: Soil Ventilation Benefited Strawberry Growth via Microbial Communities and Nutrient Cycling Under High-Density Planting
Source: Front Microbiol. 2021 Oct 18;12:666982. doi: 10.3389/fmicb.2021.666982 (PMC8558626; doi:10.3389/fmicb.2021.666982)

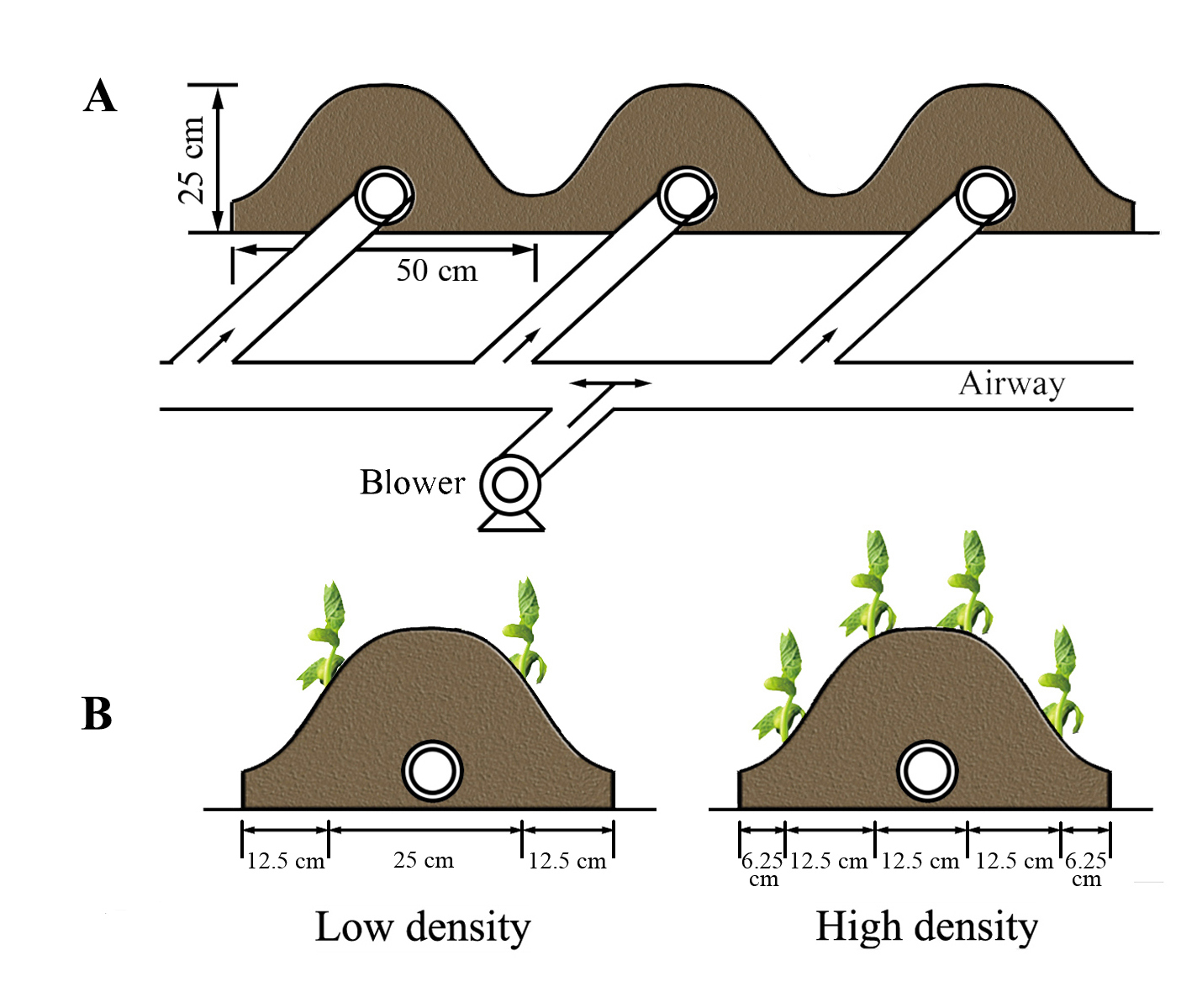

Supplement: Supplementary file 1 [file Image_1.JPEG]

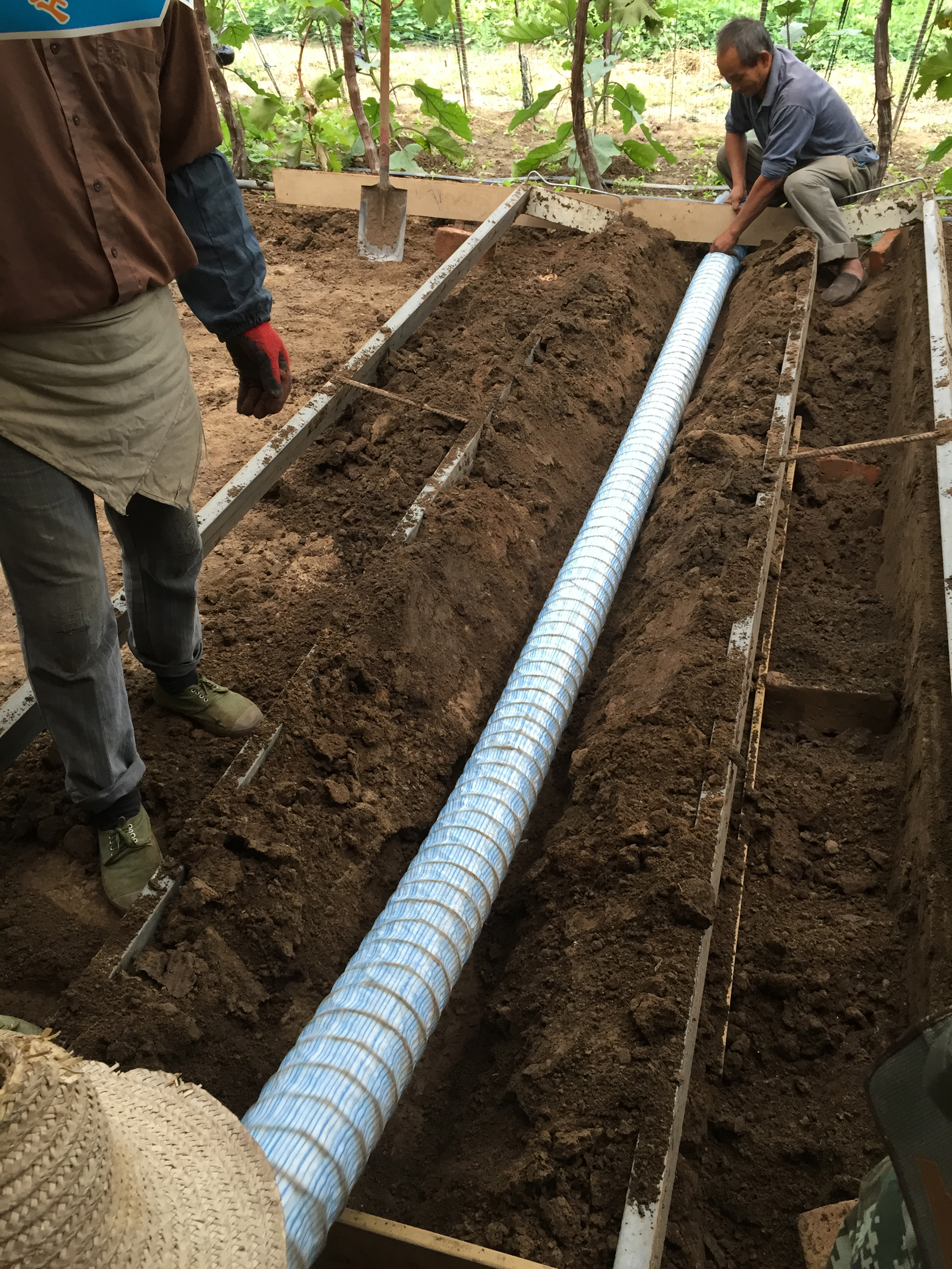

Supplement: Supplementary file 2 [file Image_2.JPEG]

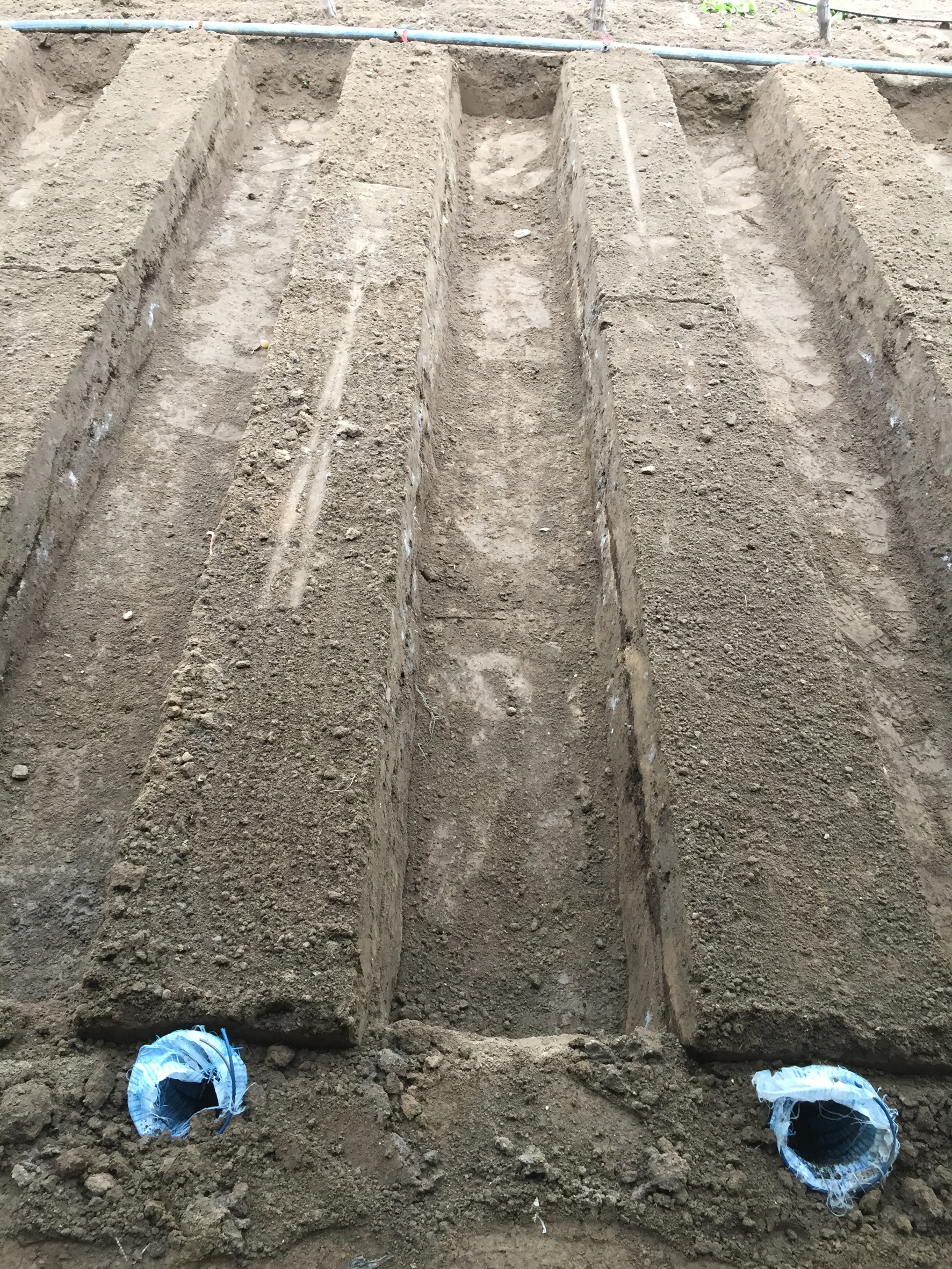

Supplement: Supplementary file 3 [file Image_3.JPEG]
