# Supplementary material for: Soil Ventilation Benefited Strawberry Growth via Microbial Communities and Nutrient Cycling Under High-Density Planting
Source: Front Microbiol. 2021 Oct 18;12:666982. doi: 10.3389/fmicb.2021.666982 (PMC8558626; doi:10.3389/fmicb.2021.666982)
Supplement: Supplementary file 4 [file Data_Sheet_1.doc]

**Supplemental tables and figures**

**Table S1 |** Three-way analysis of variance of the influence of in situ ventilation (V), density (D), and growth stage (S) and their interactions on different variables

| **Factors** | **O_2_** | |  | **CO_2_** | |  | **O_2_/CO_2_** | |  | **T** | |  | **pH** | |
| --- | --- | --- | --- | --- | --- | --- | --- | --- | --- | --- | --- | --- | --- | --- |
|  | ***F*** | ***P*** |  | ***F*** | ***P*** |  | ***F*** | ***P*** |  | ***F*** | ***P*** |  | ***F*** | ***P*** |
| **V** | 89.05 | 0.000 |  | 226.61 | 0.000 |  | 2761.76 | 0.000 |  | 190.09 | 0.000 |  | 3.44 | 0.077 |
| **D** | 3.87 | 0.068 |  | 8.52 | 0.006 |  | 182.72 | 0.000 |  | 1.08 | 0.305 |  | 0.51 | 0.507 |
| **S** | 11.61 | 0.004 |  | 7.41 | 0.014 |  | 69.30 | 0.000 |  | 3200.45 | 0.000 |  | 58.68 | 0.000 |
| **V×D** | 0.39 | 0.538 |  | 0.80 | 0.331 |  | 87.90 | 0.000 |  | 26.72 | 0.000 |  | 0.39 | 0.554 |
| **V×S** | 0.43 | 0.516 |  | 0.02 | 0.902 |  | 21.65 | 0.000 |  | 117.13 | 0.000 |  | 0.14 | 0.711 |
| **D×S** | 0.43 | 0.516 |  | 0.03 | 0.902 |  | 0.26 | 0.616 |  | 1.24 | 0.270 |  | 3.21 | 0.102 |
| **V×D×S** | 0.16 | 0.706 |  | 0.07 | 0.805 |  | 1.55 | 0.231 |  | 14.65 | 0.001 |  | 0.31 | 0.657 |

*CO_2_, soil CO_2_ concentration; D, planting density; O_2_, soil O_2_ concentration; S, growth stage; T, soil temperature; V, in situ ventilation.*

**Table S2 |** Three-way analysis of variance of the influence of in situ ventilation (V), density (D), growth stage (S), and their interactions on the studied variables

| Factors | **SOC** | |  | **SON** | |  | **TN** | |  | **TP** | |  | **TK** | |  | **AN** | |  | **AP** | |
| --- | --- | --- | --- | --- | --- | --- | --- | --- | --- | --- | --- | --- | --- | --- | --- | --- | --- | --- | --- | --- |
|  | ***F*** | ***P*** |  | ***F*** | ***P*** |  | ***F*** | ***P*** |  | ***F*** | ***P*** |  | ***F*** | ***P*** |  | ***F*** | ***P*** |  | ***F*** | ***P*** |
| **V** | 23.19 | 0.000 |  | 69.89 | 0.000 |  | 28.11 | 0.000 |  | 35.52 | 0.000 |  | 50.23 | 0.000 |  | 2.82 | 0.113 |  | 42.59 | 0.000 |
| **D** | 1.11 | 0.307 |  | 19.77 | 0.000 |  | 11.02 | 0.004 |  | 16.42 | 0.001 |  | 17.52 | 0.001 |  | 51.34 | 0.000 |  | 14.2 | 0.002 |
| **S** | 22.94 | 0.000 |  | 168.31 | 0.000 |  | 68.41 | 0.000 |  | 28.67 | 0.000 |  | 52.57 | 0.000 |  | 159 | 0.000 |  | 134.71 | 0.000 |
| **V×D** | 0.02 | 0.888 |  | 4.06 | 0.061 |  | 1.11 | 0.308 |  | 6.82 | 0.019 |  | 0.78 | 0.389 |  | 3.37 | 0.085 |  | 2.86 | 0.110 |
| **V×S** | 4.94 | 0.041 |  | 8.02 | 0.012 |  | 4.14 | 0.059 |  | 0.58 | 0.459 |  | 8.95 | 0.009 |  | 8.43 | 0.010 |  | 0.05 | 0.831 |
| **D×S** | 0.05 | 0.826 |  | 0.07 | 0.797 |  | 1.89 | 0.188 |  | 0.78 | 0.389 |  | 5.14 | 0.038 |  | 21.51 | 0.000 |  | 2.32 | 0.148 |
| **V×D×S** | 0.01 | 0.927 |  | 10.68 | 0.005 |  | 4.1 | 0.060 |  | 1.18 | 0.293 |  | 0.68 | 0.423 |  | 2.42 | 0.139 |  | 0.04 | 0.841 |
| Factors | **AK** | |  | **C/N** | |  | **INV** | |  | **CAT** | |  | **SMF** | |  | **URE** | |  |  |  |
|  | ***F*** | ***P*** |  | ***F*** | ***P*** |  | ***F*** | ***P*** |  | ***F*** | ***P*** |  | ***F*** | ***P*** |  | ***F*** | ***P*** |  |  |  |
| **V** | 35.56 | 0.000 |  | 4.87 | 0.042 |  | 62.28 | 0.000 |  | 200.3 | 0.000 |  | 142.12 | 0.000 |  | 54.01 | 0.000 |  |  |  |
| **D** | 0.01 | 0.931 |  | 5.04 | 0.039 |  | 13.72 | 0.002 |  | 0.19 | 0.669 |  | 2.39 | 0.142 |  | 8.91 | 0.009 |  |  |  |
| **S** | 601.67 | 0.000 |  | 61.87 | 0.000 |  | 32.45 | 0.000 |  | 29.53 | 0.000 |  | 24.29 | 0.000 |  | 20.09 | 0.000 |  |  |  |
| **V×D** | 0.47 | 0.503 |  | 0.05 | 0.821 |  | 0.81 | 0.382 |  | 1.06 | 0.319 |  | 1.1 | 0.310 |  | 12.62 | 0.003 |  |  |  |
| **V×S** | 1.17 | 0.295 |  | 6.88 | 0.018 |  | 0.15 | 0.701 |  | 1.38 | 0.257 |  | 1.49 | 0.240 |  | 0.00 | 0.997 |  |  |  |
| **D×S** | 5.23 | 0.036 |  | 0.01 | 0.930 |  | 1.59 | 0.226 |  | 2.93 | 0.106 |  | 0.03 | 0.865 |  | 1.84 | 0.194 |  |  |  |
| **V×D×S** | 0.91 | 0.355 |  | 0.74 | 0.401 |  | 0.65 | 0.430 |  | 18.37 | 0.001 |  | 1.05 | 0.322 |  | 0.00 | 0.946 |  |  |  |

*AN, alkaline hydrolysis nitrogen; AP, available phosphorus; AK, available potassium; C/N, soil organic carbon/total nitrogen ratio; CAT, catalase; D, planting density; INV, invertase; S, growth stage; SMF, soil ecosystem multifunctionality index; SOC, soil organic carbon; SON, soil organic N; TK, total potassium; TN, total nitrogen; TP, total phosphorus; URE, urease; V,* *ventilation.*

**Table S3 |** Three-way analysis of variance on the influence of in situ ventilation (V), density (D), growth stage (S), and their interactions on the richness and diversity indices of the microbial community

| **Factors** | **Bacteria** | | | | | | | |  | **Fungi** | | | | | | | |
| --- | --- | --- | --- | --- | --- | --- | --- | --- | --- | --- | --- | --- | --- | --- | --- | --- | --- |
|  | **Sobs** | |  | **Chao** | |  | **Shannon** | |  | **Sobs** | |  | **Chao** | |  | **Shannon** | |
|  | ***F*** | ***P*** |  | ***F*** | ***P*** |  | ***F*** | ***P*** |  | ***F*** | ***P*** |  | ***F*** | ***P*** |  | ***F*** | ***P*** |
| **V** | 0.07 | 0.800 |  | 2.33 | 0.146 |  | 0.3 | 0.594 |  | 0.07 | 0.801 |  | 1.84 | 0.194 |  | 3.7 | 0.072 |
| **D** | 10.29 | 0.005 |  | 0.16 | 0.692 |  | 9.48 | 0.007 |  | 0.79 | 0.386 |  | 5.14 | 0.038 |  | 117.33 | 0.000 |
| **S** | 6.06 | 0.026 |  | 6.95 | 0.018 |  | 2.92 | 0.107 |  | 32.91 | 0.000 |  | 0.23 | 0.640 |  | 258.2 | 0.000 |
| **V×D** | 0.78 | 0.389 |  | 1.06 | 0.319 |  | 0.61 | 0.446 |  | 3.43 | 0.083 |  | 3.71 | 0.072 |  | 84.7 | 0.000 |
| **V×S** | 0.24 | 0.630 |  | 3.2 | 0.093 |  | 0 | 0.968 |  | 1.57 | 0.228 |  | 2.42 | 0.140 |  | 20.56 | 0.000 |
| **D×S** | 10.81 | 0.005 |  | 6.57 | 0.021 |  | 2.03 | 0.173 |  | 0.97 | 0.340 |  | 2.37 | 0.143 |  | 15.12 | 0.001 |
| **V×D×S** | 0.20 | 0.661 |  | 2.3 | 0.149 |  | 5.51 | 0.032 |  | 1.83 | 0.195 |  | 1.22 | 0.285 |  | 126.05 | 0.000 |

*Sobs reflects the observed OTUs; Chao was used to evaluate community richness based on OTUs; and the Shannon index was used to assess community diversity, taking into account the contributions of rare taxa. Simpson’s index focuses on major taxa and assesses community diversity as a function of dominance.*

**Table S4a |** Three-way ANOVA on the influence of in situ ventilation (V), density (D), and growth stage (S), and their interactions on the relative abundance of the bacterial community at the phylum level

| **Bacterial**  **phyla** | **Proteobacteria** | |  | **Planctomycetes** | |  | **Acidobacteria** | |  | **Bacteroidetes** | |  | **Actinobacteria** | |  |
| --- | --- | --- | --- | --- | --- | --- | --- | --- | --- | --- | --- | --- | --- | --- | --- |
|  | ***F*** | ***P*** |  | ***F*** | ***P*** |  | ***F*** | ***P*** |  | ***F*** | ***P*** |  | ***F*** | ***P*** |  |
| **V** | 6.77 | * |  | 0.53 | 0.48 |  | 0.00 | 0.99 |  | 0.09 | 0.77 |  | 0.02 | 0.88 |  |
| **D** | 0.48 | 0.50 |  | 1.55 | 0.23 |  | 40.69 | ** |  | 10.31 | ** |  | 5.30 | * |  |
| **S** | 78.35 | ** |  | 124.76 | ** |  | 61.48 | ** |  | 0.03 | 0.86 |  | 299.63 | ** |  |
| **V×D** | 5.34 | * |  | 1.22 | 0.28 |  | 0.14 | 0.71 |  | 0.00 | 1.00 |  | 7.65 | * |  |
| **V×S** | 0.12 | 0.73 |  | 0.99 | 0.33 |  | 0.02 | 0.90 |  | 1.90 | 0.19 |  | 37.46 | ** |  |
| **D×S** | 16.83 | ** |  | 7.10 | ** |  | 4.12 | * |  | 9.62 | ** |  | 7.48 | * |  |
| **V×D×S** | 0.13 | 0.73 |  | 0.31 | 0.58 |  | 0.78 | 0.39 |  | 0.21 | 0.66 |  | 19.95 | ** |  |
| **Bacterial**  **phyla** | **Gemmatimonadetes** | |  | **Verrucomicrobia** | |  | **Chloroflexi** | |  | **Firmicutes** | |  | **Chlamydiae** | |  |
|  | ***F*** | ***P*** |  | ***F*** | ***P*** |  | ***F*** | ***P*** |  | ***F*** | ***P*** |  | ***F*** | ***P*** |  |
| **V** | 28.33 | ** |  | 3.14 | 0.10 |  | 6.04 | * |  | 21.64 | ** |  | 0.04 | 0.84 |  |
| **D** | 0.26 | 0.62 |  | 0.13 | 0.72 |  | 5.76 | * |  | 1.61 | 0.22 |  | 8.59 | ** |  |
| **S** | 1.21 | 0.29 |  | 27.94 | ** |  | 152.42 | ** |  | 187.40 | ** |  | 109.38 | ** |  |
| **V×D** | 0.70 | 0.41 |  | 0.52 | 0.48 |  | 0.03 | 0.86 |  | 0.10 | 0.76 |  | 1.86 | 0.19 |  |
| **V×S** | 1.33 | 0.27 |  | 4.66 | * |  | 15.92 | ** |  | 12.06 | ** |  | 2.95 | 0.11 |  |
| **D×S** | 5.50 | * |  | 26.22 | ** |  | 13.70 | ** |  | 0.33 | 0.57 |  | 29.77 | ** |  |
| **V×D×S** | 2.24 | 0.15 |  | 0.01 | 0.91 |  | 0.14 | 0.71 |  | 14.49 | ** |  | 4.29 | 0.05 |  |

* *P* < 0.05, ** *P* < 0.01. D, density; S, season; V, ventilation.

**Table S4b |** Three-way ANOVA on the influence of in situ ventilation (V), density (D), and growth stage (S), and their interactions on the relative abundance of the bacterial community at the class level

| **Bacterial**  **class** | **Alphaproteobacteria** | |  | **Betaproteobacteria** | |  | **Deltaproteobacteria** | |  | **Gammaproteobacteria** | |  | **Planctomycetacia** | |  | **Phycisphaerae** | |
| --- | --- | --- | --- | --- | --- | --- | --- | --- | --- | --- | --- | --- | --- | --- | --- | --- | --- |
|  | ***F*** | ***P*** |  | ***F*** | ***P*** |  | ***F*** | ***P*** |  | ***F*** | ***P*** |  | ***F*** | ***P*** |  | ***F*** | ***P*** |
| **V** | 5.34 | * |  | 13.61 | ** |  | 6.33 | * |  | 0.05 | 0.82 |  | 0.32 | 0.58 |  | 5.25 | * |
| **D** | 9.56 | ** |  | 29.60 | ** |  | 23.88 | ** |  | 9.24 | ** |  | 11.99 | ** |  | 6.47 | * |
| **S** | 0.74 | 0.40 |  | 288.34 | ** |  | 296.24 | ** |  | 9.20 | ** |  | 165.85 | ** |  | 139.81 | ** |
| **V×D** | 0.29 | 0.60 |  | 6.44 | * |  | 12.45 | ** |  | 0.13 | 0.73 |  | 0.02 | 0.90 |  | 3.02 | 0.10 |
| **V×S** | 4.42 | 0.05 |  | 0.74 | 0.40 |  | 10.93 | ** |  | 1.70 | 0.21 |  | 3.24 | 0.09 |  | 1.58 | 0.23 |
| **D×S** | 1.08 | 0.31 |  | 23.59 | ** |  | 21.89 | ** |  | 8.42 | * |  | 0.79 | 0.39 |  | 11.26 | ** |
| **V×D×S** | 2.32 | 0.15 |  | 2.04 | 0.17 |  | 10.12 | ** |  | 5.35 | * |  | 1.57 | 0.23 |  | 0.02 | 0.89 |
| **Bacterial**  **class** | **Acidobacteria** | |  | **Cytophagia** | |  | **Flavobacteriia** | |  | **Sphingobacteriia** | |  | **Actinobacteria** | |  | **Acidimicrobiia** | |
|  | ***F*** | ***P*** |  | ***F*** | ***P*** |  | ***F*** | ***P*** |  | ***F*** | ***P*** |  | ***F*** | ***P*** |  | ***F*** | ***P*** |
| **V** | 0.19 | 0.67 |  | 1.57 | 0.23 |  | 0.09 | 0.77 |  | 0.39 | 0.54 |  | 0.31 | 0.59 |  | 2.61 | 0.13 |
| **D** | 37.08 | ** |  | 0.24 | 0.63 |  | 8.12 | * |  | 46.37 | ** |  | 90.36 | ** |  | 3.51 | 0.08 |
| **S** | 75.04 | ** |  | 88.37 | ** |  | 16.80 | ** |  | 0.14 | 0.72 |  | 317.43 | ** |  | 63.15 | ** |
| **V×D** | 0.53 | 0.48 |  | 0.31 | 0.58 |  | 0.36 | 0.56 |  | 7.08 | * |  | 1.68 | 0.21 |  | 16.57 | ** |
| **V×S** | 0.10 | 0.75 |  | 15.19 | ** |  | 0.27 | 0.61 |  | 0.60 | 0.45 |  | 41.09 | ** |  | 17.28 | ** |
| **D×S** | 8.36 | * |  | 0.45 | 0.51 |  | 14.28 | ** |  | 1.65 | 0.22 |  | 1.43 | 0.25 |  | 38.74 | ** |
| **V×D×S** | 0.93 | 0.35 |  | 4.84 | * |  | 0.37 | 0.55 |  | 0.04 | 0.85 |  | 31.39 | ** |  | 10.58 | ** |
| **Bacterial**  **class** | **Gemmatimonadetes** | |  | **Verrucomicrobiae** | |  | **Bacilli** | |  | **OM190** | |  |  | |  |  | |
|  | ***F*** | ***P*** |  | ***F*** | ***P*** |  | ***F*** | ***P*** |  | ***F*** | ***P*** |  |  |  |  |  |  |
| **V** | 28.33 | ** |  | 0.32 | 0.58 |  | 21.50 | ** |  | 11.23 | ** |  |  |  |  |  |  |
| **D** | 0.26 | 0.62 |  | 12.76 | ** |  | 1.17 | 0.30 |  | 0.88 | 0.36 |  |  |  |  |  |  |
| **S** | 1.21 | 0.29 |  | 124.52 | ** |  | 169.43 | ** |  | 174.08 | ** |  |  |  |  |  |  |
| **V×D** | 0.70 | 0.41 |  | 8.59 | ** |  | 0.06 | 0.82 |  | 5.13 | * |  |  |  |  |  |  |
| **V×S** | 1.33 | 0.27 |  | 29.10 | ** |  | 8.83 | * |  | 16.61 | ** |  |  |  |  |  |  |
| **D×S** | 5.50 | * |  | 39.46 | ** |  | 0.08 | 0.78 |  | 24.82 | ** |  |  |  |  |  |  |
| **V×D×S** | 2.24 | 0.15 |  | 0.74 | 0.40 |  | 15.34 | ** |  | 3.39 | 0.08 |  |  |  |  |  |  |

* *P* < 0.05, ** *P* < 0.01. D, density; S, season; V, ventilation.

**Table S4c |** Three-way ANOVA on the influence of in situ ventilation (V), density (D), and growth stage (S), and their interactions on the relative abundance of the bacterial community at the family level

| **Bacterial family** | **Erythrobacteraceae** | |  | **Xanthomonadaceae** | |  | **Pseudomonadaceae** | |  | **Planctomycetaceae** | |  | **Phycisphaeraceae** | |  | **Cytophagaceae** | |
| --- | --- | --- | --- | --- | --- | --- | --- | --- | --- | --- | --- | --- | --- | --- | --- | --- | --- |
|  | ***F*** | ***P*** |  | ***F*** | ***P*** |  | ***F*** | ***P*** |  | ***F*** | ***P*** |  | ***F*** | ***P*** |  | ***F*** | ***P*** |
| **V** | 0.43 | 0.52 |  | 0.79 | 0.39 |  | 1.76 | 0.20 |  | 0.32 | 0.58 |  | 7.65 | * |  | 2.59 | 0.13 |
| **D** | 145.81 | ** |  | 60.84 | ** |  | 8.49 | * |  | 11.99 | ** |  | 69.43 | ** |  | 1.17 | 0.30 |
| **S** | 1181.73 | ** |  | 412.04 | ** |  | 23.03 | ** |  | 165.85 | ** |  | 167.16 | ** |  | 90.12 | ** |
| **V×D** | 0.01 | 0.91 |  | 3.12 | 0.10 |  | 0.02 | 0.89 |  | 0.02 | 0.90 |  | 5.75 | * |  | 0.26 | 0.62 |
| **V×S** | 8.00 | * |  | 0.01 | 0.90 |  | 0.43 | 0.52 |  | 3.24 | 0.09 |  | 2.44 | 0.14 |  | 15.09 | ** |
| **D×S** | 4.27 | 0.06 |  | 31.73 | ** |  | 5.85 | * |  | 0.79 | 0.39 |  | 19.55 | ** |  | 1.39 | 0.26 |
| **V×D×S** | 12.89 | ** |  | 0.01 | 0.90 |  | 11.13 | ** |  | 1.57 | 0.23 |  | 1.43 | 0.25 |  | 3.43 | 0.08 |
| **Bacterial family** | **Flavobacteriaceae** | |  | **Sphingomonadaceae** | |  | **Chitinophagaceae** | |  | **Gemmatimonadaceae** | |  | **Verrucomicrobiaceae** | |  | **Bacillaceae** | |
|  | ***F*** | ***P*** |  | ***F*** | ***P*** |  | ***F*** | ***P*** |  | ***F*** | ***P*** |  | ***F*** | ***P*** |  | ***F*** | ***P*** |
| **V** | 0.18 | 0.68 |  | 4.48 | 0.05 |  | 1.47 | 0.24 |  | 25.35 | ** |  | 0.15 | 0.71 |  | 31.88 | ** |
| **D** | 8.09 | * |  | 3.09 | 0.10 |  | 24.13 | ** |  | 1.26 | 0.28 |  | 15.01 | ** |  | 0.89 | 0.36 |
| **S** | 18.72 | ** |  | 227.60 | ** |  | 6.20 | * |  | 0.68 | 0.42 |  | 132.72 | ** |  | 161.29 | ** |
| **V×D** | 0.44 | 0.52 |  | 3.19 | 0.09 |  | 0.79 | 0.39 |  | 1.30 | 0.27 |  | 8.11 | * |  | 0.32 | 0.58 |
| **V×S** | 0.33 | 0.57 |  | 3.29 | 0.09 |  | 0.04 | 0.84 |  | 2.07 | 0.17 |  | 33.33 | ** |  | 6.16 | * |
| **D×S** | 13.01 | ** |  | 6.53 | * |  | 0.00 | 0.95 |  | 2.31 | 0.15 |  | 40.16 | ** |  | 0.00 | 0.99 |
| **V×D×S** | 0.60 | 0.45 |  | 9.81 | ** |  | 1.15 | 0.30 |  | 2.88 | 0.11 |  | 1.85 | 0.19 |  | 15.24 | ** |

* *P* < 0.05, ** *P* < 0.01. D, density; S, season; V, ventilation.

**Table S4d |** Three-way ANOVA on the influence of in situ ventilation (V), density (D), and growth stage (S), and their interactions on the relative abundance of the bacterial community at the genus level

| **Bacterial genus** | **Arenimonas** | |  | **Pseudomonas** | |  | **Planctomyces** | |  | **Bryobacter** | |  | **Blastocatella** | |
| --- | --- | --- | --- | --- | --- | --- | --- | --- | --- | --- | --- | --- | --- | --- |
|  | ***F*** | ***P*** |  | ***F*** | ***P*** |  | ***F*** | ***P*** |  | ***F*** | ***P*** |  | ***F*** | ***P*** |
| **V** | 6.84 | * |  | 1.82 | 0.20 |  | 0.01 | 0.94 |  | 1.83 | 0.20 |  | 21.90 | ** |
| **D** | 144.80 | ** |  | 18.15 | ** |  | 1.23 | 0.28 |  | 1.51 | 0.24 |  | 84.90 | ** |
| **S** | 117.67 | ** |  | 32.66 | ** |  | 154.03 | ** |  | 50.12 | ** |  | 1336.90 | ** |
| **V×D** | 16.09 | ** |  | 0.41 | 0.53 |  | 0.18 | 0.68 |  | 8.79 | ** |  | 0.02 | 0.89 |
| **V×S** | 0.10 | 0.76 |  | 0.37 | 0.55 |  | 3.22 | 0.09 |  | 2.05 | 0.17 |  | 0.77 | 0.39 |
| **D×S** | 26.29 | ** |  | 14.78 | ** |  | 3.94 | 0.06 |  | 0.39 | 0.54 |  | 161.90 | ** |
| **V×D×S** | 0.06 | 0.81 |  | 17.15 | ** |  | 3.09 | 0.10 |  | 0.25 | 0.62 |  | 17.99 | ** |
| **Bacterial genus** | **Chryseolinea** | |  | **Flavobacterium** | |  | **Sphingomonas** | |  | **Gemmatimonas** | |  | **Bacillus** | |
|  | ***F*** | ***P*** |  | ***F*** | ***P*** |  | ***F*** | ***P*** |  | ***F*** | ***P*** |  | ***F*** | ***P*** |
| **V** | 0.87 | 0.37 |  | 0.04 | 0.84 |  | 7.37 | * |  | 41.51 | ** |  | 29.70 | ** |
| **D** | 2.14 | 0.16 |  | 9.63 | ** |  | 7.90 | * |  | 2.56 | 0.13 |  | 0.77 | 0.39 |
| **S** | 90.92 | ** |  | 13.92 | ** |  | 203.64 | ** |  | 18.13 | ** |  | 137.31 | ** |
| **V×D** | 1.00 | 0.33 |  | 0.40 | 0.54 |  | 1.93 | 0.18 |  | 0.93 | 0.35 |  | 0.94 | 0.35 |
| **V×S** | 18.70 | ** |  | 0.04 | 0.84 |  | 8.25 | * |  | 3.55 | 0.08 |  | 5.58 | * |
| **D×S** | 0.31 | 0.59 |  | 19.48 | ** |  | 9.57 | ** |  | 5.12 | * |  | 0.04 | 0.83 |
| **V×D×S** | 1.54 | 0.23 |  | 0.31 | 0.59 |  | 11.84 | ** |  | 1.56 | 0.23 |  | 13.65 | ** |

* *P* < 0.05, ** *P* < 0.01. D, density; S, season; V, ventilation.

**Table S5 |** Influence of in situ ventilation (V), density (D), and growth stage (S) and their interactions on the structure of total microbial communities by PERMANOVA

| **Bacteria** |  | **Phylum** | |  | **Class** | |  | **Family** | |  | **Genus** | |
| --- | --- | --- | --- | --- | --- | --- | --- | --- | --- | --- | --- | --- |
|  |  | ***F*** | ***P*** |  | ***F*** | ***P*** |  | ***F*** | ***P*** |  | ***F*** | ***P*** |
| **V** |  | 1.24 | 0.281 |  | 5.63 | 0.031 |  | 14.07 | 0.002 |  | 9.93 | 0.006 |
| **D** |  | 7.67 | 0.014 |  | 10.95 | 0.004 |  | 7.74 | 0.013 |  | 43.79 | 0.000 |
| **S** |  | 2.01 | 0.175 |  | 2.27 | 0.152 |  | 5.95 | 0.027 |  | 85.94 | 0.000 |
| **V×D** |  | 9.66 | 0.007 |  | 0.07 | 0.795 |  | 3.47 | 0.081 |  | 4.34 | 0.054 |
| **V×S** |  | 0.02 | 0.894 |  | 4.63 | 0.047 |  | 14.43 | 0.002 |  | 26.20 | 0.000 |
| **D×S** |  | 7.12 | 0.017 |  | 1.40 | 0.254 |  | 7.40 | 0.015 |  | 2.82 | 0.112 |
| **V×D×S** |  | 2.97 | 0.104 |  | 0.45 | 0.511 |  | 5.39 | 0.034 |  | 4.45 | 0.051 |
| **Fungi** |  | **Phylum** | |  | **Class** | |  | **Family** | |  | **Genus** | |
|  |  | ***F*** | ***P*** |  | ***F*** | ***P*** |  | ***F*** | ***P*** |  | ***F*** | ***P*** |
| **V** |  | 0.46 | 0.509 |  | 0.81 | 0.381 |  | 2.80 | 0.113 |  | 1.53 | 0.235 |
| **D** |  | 9.27 | 0.008 |  | 8.33 | 0.011 |  | 6.00 | 0.026 |  | 6.46 | 0.022 |
| **S** |  | 297.29 | 0.000 |  | 291.25 | 0.000 |  | 257.98 | 0.000 |  | 236.06 | 0.000 |
| **V×D** |  | 0.42 | 0.524 |  | 0.47 | 0.504 |  | 0.12 | 0.731 |  | 0.06 | 0.809 |
| **V×S** |  | 13.26 | 0.002 |  | 12.91 | 0.002 |  | 12.36 | 0.003 |  | 10.96 | 0.004 |
| **D×S** |  | 78.14 | 0.000 |  | 81.59 | 0.000 |  | 102.16 | 0.000 |  | 84.30 | 0.000 |
| **V×D×S** |  | 20.11 | 0.000 |  | 23.83 | 0.000 |  | 30.43 | 0.000 |  | 29.28 | 0.000 |

*D, planting density; S, growth stage; V, in situ ventilation.*

**Table S6 |** Three-way ANOVA on the influence of in situ ventilation (V), density (D), and growth stage (S), and their interactions on the relative abundance of the fungal community at the phylum, class, family and genus levels

| **Fungi** | **Phylum** | | | | | | | | | | | | | | | | | |  | **Class** | | | | | | | | |
| --- | --- | --- | --- | --- | --- | --- | --- | --- | --- | --- | --- | --- | --- | --- | --- | --- | --- | --- | --- | --- | --- | --- | --- | --- | --- | --- | --- | --- |
|  | **Ascomycota** | | |  | **Basidiomycota** | | | | |  | **Chytridiomycota** | | |  | **Zygomycota** | |  | **Eurotiomycetes** | | | |  | **Pezizomycetes** | |  | **Agaricomycetes** | |  |
|  | ***F*** | ***P*** | |  | ***F*** | | | ***P*** | |  | ***F*** | ***P*** | |  | ***F*** | ***P*** |  | ***F*** | | | ***P*** |  | ***F*** | ***P*** |  | ***F*** | ***P*** |  |
| **V** | 1.70 | 0.21 | |  | 35.78 | | | ** | |  | 10.64 | ** | |  | 0.01 | 0.93 |  | 3.89 | | | 0.07 |  | 11.40 | ** |  | 35.15 | ** |  |
| **D** | 4.94 | * | |  | 72.40 | | | ** | |  | 2.01 | 0.18 | |  | 7.26 | * |  | 11.13 | | | ** |  | 5.46 | * |  | 70.79 | ** |  |
| **S** | 102.93 | ** | |  | 73.43 | | | ** | |  | 77.79 | ** | |  | 275.98 | ** |  | 95.19 | | | ** |  | 5.20 | * |  | 72.66 | ** |  |
| **V×D** | 21.41 | ** | |  | 75.68 | | | ** | |  | 0.52 | 0.48 | |  | 20.60 | ** |  | 14.04 | | | ** |  | 21.17 | ** |  | 74.12 | ** |  |
| **V×S** | 0.03 | 0.88 | |  | 102.68 | | | ** | |  | 1.44 | 0.25 | |  | 7.00 | * |  | 2.19 | | | 0.16 |  | 0.09 | 0.77 |  | 101.52 | ** |  |
| **D×S** | 18.14 | ** | |  | 117.97 | | | ** | |  | 26.23 | ** | |  | 31.52 | ** |  | 26.86 | | | ** |  | 1.64 | 0.22 |  | 116.39 | ** |  |
| **V×D×S** | 0.21 | 0.65 | |  | 5.69 | | | 0.03 | |  | 2.32 | 0.15 | |  | 14.42 | ** |  | 5.45 | | | * |  | 0.11 | 0.75 |  | 5.70 | * |  |
| **Fungi** | **Family** | | | | | | | | |  | **Genus** | | | | | | | |  | | | | | | | | | |
|  | **Trichocomaceae** | | | | |  | **Aspergillus** | | |  | **Lobulomyces** | |  | **Triparticalcar** | | | | |  | | | | | | | | | |
|  | ***F*** | | ***P*** | | |  | ***F*** | | ***P*** |  | ***F*** | ***P*** |  | ***F*** | | ***P*** | | |  | | | | | | | | | |
| **V** | 3.06 | | 0.10 | | |  | 3.18 | | 0.09 |  | 5.79 | * |  | 2.70 | | 0.12 | | |  | | | | | | | | | |
| **D** | 19.47 | | ** | | |  | 20.40 | | ** |  | 1.73 | 0.21 |  | 2.40 | | 0.14 | | |  | | | | | | | | | |
| **S** | 83.24 | | ** | | |  | 84.65 | | ** |  | 24.81 | ** |  | 35.05 | | ** | | |  | | | | | | | | | |
| **V×D** | 15.80 | | ** | | |  | 16.97 | | ** |  | 4.69 | * |  | 3.12 | | 0.10 | | |  | | | | | | | | | |
| **V×S** | 2.14 | | 0.16 | | |  | 1.92 | | 0.18 |  | 6.70 | * |  | 2.03 | | 0.17 | | |  | | | | | | | | | |
| **D×S** | 32.20 | | ** | | |  | 34.99 | | ** |  | 1.36 | 0.26 |  | 3.23 | | 0.09 | | |  | | | | | | | | | |
| **V×D×S** | 5.94 | | * | | |  | 6.33 | | * |  | 0.51 | 0.49 |  | 3.57 | | 0.08 | | |  | | | | | | | | | |

* *P* < 0.05, ** *P* < 0.01. D, density; S, season; V, ventilation.

**Table S7a |** Three-way ANOVA on the influence of in situ ventilation (V), density (D), and growth stage (S), and their interactions on functions related to the soil C cycle from bacterial communities

| **C**  **cycle** | **Chemoheterotrophy** | |  | **Aerobic chemoheterotrophy** | |  | **Predatory or exoparasitic** | |  | **Intracellular parasites** | |  | **Phototrophy** | |
| --- | --- | --- | --- | --- | --- | --- | --- | --- | --- | --- | --- | --- | --- | --- |
|  | ***F*** | ***P*** |  | ***F*** | ***P*** |  | ***F*** | ***P*** |  | ***F*** | ***P*** |  | ***F*** | ***P*** |
| **V** | 0.02 | 0.89 |  | 0.03 | 0.86 |  | 4.57 | * |  | 0.67 | 0.43 |  | 0.72 | 0.41 |
| **D** | 25.63 | ** |  | 44.37 | ** |  | 1.71 | 0.21 |  | 27.20 | ** |  | 0.34 | 0.57 |
| **S** | 65.47 | ** |  | 107.21 | ** |  | 157.75 | ** |  | 80.52 | ** |  | 31.28 | ** |
| **V×D** | 0.21 | 0.65 |  | 0.40 | 0.54 |  | 5.26 | * |  | 0.80 | 0.38 |  | 0.72 | 0.41 |
| **V×S** | 1.81 | 0.20 |  | 2.01 | 0.18 |  | 15.03 | ** |  | 0.01 | 0.92 |  | 0.00 | 0.96 |
| **D×S** | 10.00 | ** |  | 16.78 | ** |  | 22.47 | ** |  | 29.51 | ** |  | 1.54 | 0.23 |
| **V×D×S** | 2.55 | 0.13 |  | 3.31 | 0.09 |  | 10.12 | ** |  | 0.45 | 0.51 |  | 0.13 | 0.72 |
| **C**  **cycle** | **Fermentation** | |  | **Chitinolysis** | |  | **Photoautotrophy** | |  | **Cyanobacteria** | |  | **Oxygenic photoautotrophy** | |
|  | ***F*** | ***P*** |  | ***F*** | ***P*** |  | ***F*** | ***P*** |  | ***F*** | ***P*** |  | ***F*** | ***P*** |
| **V** | 0.39 | 0.54 |  | 0.03 | 0.87 |  | 0.77 | 0.39 |  | 0.59 | 0.45 |  | 0.59 | 0.45 |
| **D** | 1.87 | 0.19 |  | 0.03 | 0.85 |  | 6.56 | * |  | 5.87 | * |  | 5.87 | * |
| **S** | 7.81 | * |  | 72.50 | ** |  | 20.55 | ** |  | 26.30 | ** |  | 26.30 | ** |
| **V×D** | 0.15 | 0.70 |  | 6.16 | * |  | 4.03 | 0.06 |  | 4.40 | 0.05 |  | 4.40 | 0.05 |
| **V×S** | 2.08 | 0.17 |  | 0.58 | 0.46 |  | 0.44 | 0.52 |  | 0.82 | 0.38 |  | 0.82 | 0.38 |
| **D×S** | 0.51 | 0.48 |  | 20.51 | ** |  | 2.71 | 0.12 |  | 2.26 | 0.15 |  | 2.26 | 0.15 |
| **V×D×S** | 0.72 | 0.41 |  | 4.71 | * |  | 2.96 | 0.10 |  | 1.94 | 0.18 |  | 1.94 | 0.18 |
| **C cycle** | **Aromatic compound degradation** | |  | **Photoheterotrophy** | |  | **Methylotrophy** | |  | **Chloroplasts** | |  |  | |
|  | ***F*** | ***P*** |  | ***F*** | ***P*** |  | ***F*** | ***P*** |  | ***F*** | ***P*** |  |  |  |
| **V** | 0.37 | 0.55 |  | 0.39 | 0.54 |  | 10.55 | * |  | 0.55 | 0.47 |  |  |  |
| **D** | 25.18 | ** |  | 2.89 | 0.11 |  | 2.79 | 0.11 |  | 1.54 | 0.23 |  |  |  |
| **S** | 68.50 | ** |  | 16.95 | 0.00 |  | 179.55 | ** |  | 14.56 | ** |  |  |  |
| **V×D** | 0.40 | 0.53 |  | 0.76 | 0.40 |  | 0.96 | 0.34 |  | 0.12 | 0.73 |  |  |  |
| **V×S** | 2.89 | 0.11 |  | 1.25 | 0.28 |  | 11.38 | ** |  | 0.01 | 0.92 |  |  |  |
| **D×S** | 5.06 | * |  | 0.26 | 0.62 |  | 32.57 | ** |  | 0.81 | 0.38 |  |  |  |
| **V×D×S** | 2.25 | 0.15 |  | 0.87 | 0.36 |  | 0.96 | 0.34 |  | 0.65 | 0.43 |  |  |  |

* *P* < 0.05, ** *P* < 0.01. D, density; S, season; V, ventilation.

.

**Table S7b |** Three-way ANOVA on the influence of in situ ventilation (V), density (D), and growth stage (S), and their interactions on functions related to the soil N cycle from bacterial communities

| **N**  **cycle** | **nitrate reduction** | |  | **ureolysis** | |  | **nitrification** | |  | **aerobic nitrite oxidation** | |  | **nitrate respiration** | |  | **aerobic ammonia oxidation** | |
| --- | --- | --- | --- | --- | --- | --- | --- | --- | --- | --- | --- | --- | --- | --- | --- | --- | --- |
|  | ***F*** | ***P*** |  | ***F*** | ***P*** |  | ***F*** | ***P*** |  | ***F*** | ***P*** |  | ***F*** | ***P*** |  | ***F*** | ***P*** |
| **V** | 0.04 | 0.84 |  | 29.28 | ** |  | 0.01 | 0.91 |  | 0.89 | 0.36 |  | 0.01 | 0.91 |  | 0.89 | 0.36 |
| **D** | 0.24 | 0.63 |  | 49.38 | ** |  | 5.79 | * |  | 1.83 | 0.19 |  | 13.73 | ** |  | 1.83 | 0.19 |
| **S** | 26.94 | ** |  | 218.96 | ** |  | 91.05 | ** |  | 10.49 | ** |  | 36.83 | ** |  | 10.49 | ** |
| **V×D** | 0.56 | 0.47 |  | 27.10 | ** |  | 7.00 | * |  | 0.20 | 0.66 |  | 6.00 | * |  | 0.20 | 0.66 |
| **V×S** | 2.53 | 0.13 |  | 5.81 | * |  | 4.41 | 0.05 |  | 12.14 | ** |  | 6.62 | * |  | 12.14 | ** |
| **D×S** | 0.03 | 0.86 |  | 6.71 | * |  | 9.55 | ** |  | 0.49 | 0.50 |  | 0.95 | 0.35 |  | 0.49 | 0.50 |
| **V×D×S** | 0.29 | 0.60 |  | 2.53 | 0.13 |  | 7.74 | * |  | 6.67 | * |  | 0.76 | 0.40 |  | 6.67 | * |
| **N**  **cycle** | **nitrogen fixation** | |  | **nitrite respiration** | |  | **nitrate denitrification** | |  | **nitrite denitrification** | |  | **nitrous oxide denitrification** | |  | **denitrification** | |
|  | ***F*** | ***P*** |  | ***F*** | ***P*** |  | ***F*** | ***P*** |  | ***F*** | ***P*** |  | ***F*** | ***P*** |  | ***F*** | ***P*** |
| **V** | 10.15 | 0.01 |  | 8.34 | * |  | 0.48 | 0.50 |  | 0.48 | 0.50 |  | 0.48 | 0.50 |  | 0.48 | 0.50 |
| **D** | 51.99 | ** |  | 83.52 | ** |  | 2.84 | 0.11 |  | 2.84 | 0.11 |  | 2.84 | 0.11 |  | 2.84 | 0.11 |
| **S** | 10.15 | ** |  | 16.20 | ** |  | 0.16 | 0.70 |  | 0.16 | 0.70 |  | 0.16 | 0.70 |  | 0.16 | 0.70 |
| **V×D** | 8.61 | ** |  | 21.98 | ** |  | 0.16 | 0.70 |  | 0.16 | 0.70 |  | 0.16 | 0.70 |  | 0.16 | 0.70 |
| **V×S** | 9.62 | ** |  | 0.81 | 0.38 |  | 0.48 | 0.50 |  | 0.48 | 0.50 |  | 0.48 | 0.50 |  | 0.48 | 0.50 |
| **D×S** | 7.66 | * |  | 2.75 | 0.12 |  | 1.66 | 0.22 |  | 1.66 | 0.22 |  | 1.66 | 0.22 |  | 1.66 | 0.22 |
| **V×D×S** | 4.75 | * |  | 3.77 | 0.07 |  | 3.93 | 0.06 |  | 3.93 | 0.06 |  | 3.93 | 0.06 |  | 3.93 | 0.06 |

* *P* < 0.05, ** *P* < 0.01. D, density; S, season; V, ventilation.

**Table S8 |** Three-way ANOVA on the influence of in situ ventilation (V), density (D), and growth stage (S), and their interactions on functions related to fungal communities

| **Factors** | **Plant Pathogen** | |  | **Fungal Parasite** | |  | **Soil Saprotroph** | |  | **Litter Saprotroph** | |
| --- | --- | --- | --- | --- | --- | --- | --- | --- | --- | --- | --- |
|  | ***F*** | ***P*** |  | ***F*** | ***P*** |  | ***F*** | ***P*** |  | ***F*** | ***P*** |
| **V** | 0.02 | 0.88 |  | 11.88 | ** |  | 19.56 | ** |  | 12.60 | ** |
| **D** | 9.50 | ** |  | 9.24 | ** |  | 10.91 | ** |  | 8.53 | * |
| **S** | 55.64 | ** |  | 106.46 | ** |  | 98.60 | ** |  | 61.62 | ** |
| **V×D** | 3.48 | 0.08 |  | 12.76 | ** |  | 0.43 | 0.52 |  | 0.88 | 0.36 |
| **V×S** | 1.04 | 0.32 |  | 11.98 | ** |  | 0.59 | 0.45 |  | 1.15 | 0.30 |
| **D×S** | 0.45 | 0.51 |  | 33.85 | ** |  | 0.04 | 0.84 |  | 1.16 | 0.30 |
| **V×D×S** | 0.01 | 0.92 |  | 78.81 | ** |  | 5.36 | * |  | 11.92 | ** |
| **Factors** | **Dung Saprotroph** | |  | **Wood Saprotroph** | |  | **Clavicipitaceous Endophyte** | |  |  | |
|  | ***F*** | ***P*** |  | ***F*** | ***P*** |  | ***F*** | ***P*** |  |  |  |
| **V** | 2.25 | 0.15 |  | 0.00 | 0.99 |  | 0.25 | 0.62 |  |  |  |
| **D** | 7.41 | * |  | 4.00 | 0.06 |  | 0.19 | 0.67 |  |  |  |
| **S** | 143.67 | ** |  | 154.97 | ** |  | 69.48 | ** |  |  |  |
| **V×D** | 3.51 | 0.08 |  | 1.62 | 0.22 |  | 0.20 | 0.66 |  |  |  |
| **V×S** | 0.07 | 0.79 |  | 0.16 | 0.70 |  | 1.24 | 0.28 |  |  |  |
| **D×S** | 0.30 | 0.59 |  | 0.53 | 0.48 |  | 0.32 | 0.58 |  |  |  |
| **V×D×S** | 0.41 | 0.53 |  | 0.25 | 0.62 |  | 0.40 | 0.54 |  |  |  |

* *P* < 0.05, ** *P* < 0.01. D, density; S, season; V, ventilation.

**Supplemental figures**

**Fig.S1** Schematics of strawberry planting patterns under different in situ ventilation treatments

**Fig.S2** Photo of ventilation pipe line.

**Fig.S3** Photo of planting row after buried pipe.
